# AI‐Guided Inverse Design and Discovery of Recyclable Vitrimeric Polymers

**DOI:** 10.1002/advs.202411385

**Published:** 2024-12-16

**Authors:** Yiwen Zheng, Prakash Thakolkaran, Agni K. Biswal, Jake A. Smith, Ziheng Lu, Shuxin Zheng, Bichlien H. Nguyen, Siddhant Kumar, Aniruddh Vashisth

**Affiliations:** ^1^ Department of Mechanical Engineering University of Washington Seattle WA 98195 USA; ^2^ Department of Materials Science and Engineering Delft University of Technology Delft CD 2628 The Netherlands; ^3^ Microsoft Research Redmond WA 98052 USA; ^4^ Paul G. Allen School of Computer Science and Engineering University of Washington Seattle WA 98195 USA; ^5^ Microsoft Research Asia Beijing 100080 China

**Keywords:** generative models, machine learning, molecular dynamics, materials design, recyclable polymers, vitrimers

## Abstract

Vitrimer is a new, exciting class of sustainable polymers with healing abilities due to their dynamic covalent adaptive networks. However, a limited choice of constituent molecules restricts their property space and potential applications. To overcome this challenge, an innovative approach coupling molecular dynamics (MD) simulations and a novel graph variational autoencoder (VAE) model for inverse design of vitrimer chemistries with desired glass transition temperature (*T*_g_) is presented. The first diverse vitrimer dataset of one million chemistries is curated and *T*_g_ for 8,424 of them is calculated by high‐throughput MD simulations calibrated by a Gaussian process model. The proposed VAE employs dual graph encoders and a latent dimension overlapping scheme which allows for individual representation of multi‐component vitrimers. High accuracy and efficiency of the framework are demonstrated by discovering novel vitrimers with desirable *T*_g_ beyond the training regime. To validate the effectiveness of the framework in experiments, vitrimer chemistries are generated with a target *T*_g_ = 323 K. By incorporating chemical intuition, a novel vitrimer with *T*_g_ of 311–317 K is synthesized, experimentally demonstrating healability and flowability. The proposed framework offers an exciting tool for polymer chemists to design and synthesize novel, sustainable polymers for various applications.

## Graphical abstract

This study presents a molecular dynamics (MD) ‐ machine learning (ML) framework for inverse design of vitrimers with targeted glass transition temperature (*T*_g_). A variational autoencoder model with dual graph encoders is trained using a vitrimer dataset constructed by MD simulations. The efficacy of the framework is demonstrated by the discovery of vitrimers with *T*_g_ beyond the training regime and experimental synthesis of a novel vitrimer chemistry.

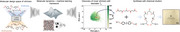

## 1 Introduction

Polymers are essential to a broad range of applications from cars and wind turbines to smartphones, medical devices and more; however their performance decreases over their life‐cycle initiated by bond breaking at the molecular scale due to high stress, oxidation, or other factors. Mechanical damage due to the rupturing of covalent bonds in traditional thermosets and thermoplastics is irreversible, resulting in crack formation and finally failure.^[^ ^1^ ^]^ In such circumstances, plastics end up in waste streams due to the inability to serve the desired purpose, which presents two key challenges for sustainability. First, failure in repairing mechanical damage means polymer parts and often entire assemblies must be replaced, resulting in high economic cost and further increasing the 430 million tons of plastic produced annually. Second, the inability of polymers to repair molecular damage poses a fundamental challenge preventing the recycling of thermosets altogether and the degradation of thermoplastics such as polyethylene terephthalate (PET) water bottles into highly degraded secondary raw materials.

**Figure 1:**
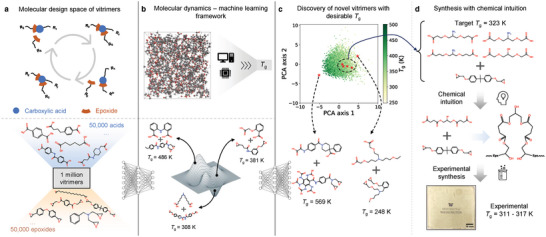
Schematic overview of this work. a) A transesterification vitrimer comprises a carboxylic acid and an epoxide. The reversible covalent bond between acid and epoxide allows them to detach from and attach to each other, thus healing the polymer. The design space for vitrimers is defined as all possible combinations of 50 000 carboxylic acids and 50 000 epoxides and a vitrimer dataset is built by sampling from the design space. b) We use calibrated MD simulations to calculate *T*_g_ on a subset of vitrimers. The vitrimer dataset and *T*_g_ are inputs to the VAE model. c) By optimizing latent vectors according to desirable *T*_g_, novel vitrimers with *T*_g_ = 569 K and 248 K are discovered. d) Synthesis of novel vitrimer chemistry proposed by the framework for target *T*_g_ of 323 K (50 °C).

Healable polymers, particularly a new class called vitrimers, offer a potential solution to the plastic waste problem. Combining durability with end‐of‐life recyclability, vitrimers have the potential to greatly reduce the amount of plastic production and waste.^[^ ^2^ ^]^ The defining molecular feature of vitrimers is an associative dynamic covalent adaptive network (CAN) which allows the constituents of polymer chains to attach to and detach from each other while conserving crosslinking density under an external stimulus such as heat. This gives vitrimers the ability to self‐healing without loss of viscosity^[^ ^3^ ^]^ (**Figure** **1**a). This exchange of constituents is termed a rearrangement reaction, and polymer scientists have found multiple reaction chemistries on which to base vitrimers, including transesterification, disulfide exchange, and imine exchange.^[^ ^4^ ^]^ However, available vitrimers have restricted thermo‐mechanical properties due to limited commercially available monomers (i.e., building blocks) for synthesizing these polymers, which is a key impediment to widespread applications of vitrimers.

The structure‐property relationships of polymers have been primarily investigated in a forward manner: given a set of polymers, one queries their properties by experiments and simulations.^[^ ^5^, ^6^ ^]^ At the early stage, most of the novel polymers are discovered and synthesized based on chemical intuition in a trial‐and‐error fashion.^[^ ^7^ ^]^ As chemical synthesis of polymers is expensive and time‐consuming, virtual specimen fabrication and characterization of desired chemical structures using molecular dynamics (MD) simulations may be employed to reduce the cost of experimentation. MD is a simulation technique situated at the interface of quantum mechanics and classical mechanics and has been widely employed to assist the discovery process.^[^ ^8^ ^]^ Virtual characterization using MD has helped in gaining insights about the effect of polymer molecular structures on mechanical properties,^[^ ^9^ ^]^ glass transition temperature^[^ ^10^ ^]^ and self‐healing.^[^ ^11^, ^12^ ^]^ However, scaled computational screens assisted by MD or other simulation methods remain costly, even with the development of high‐performance computing.^[^ ^13^ ^]^ As a result, the searchable design space is limited to the order of 10^3^ to 10^5^ compositions.

Advances in machine learning (ML) algorithms offer an opportunity to accelerate polymer discovery by learning from available data, revealing hidden patterns in material properties^[^ ^14^ ^]^ and reducing the need for costly experiments and simulations.^[^ ^15^ ^]^ Various ML methods have been employed to design organic molecules and polymers, including forward predictive models,^[^ ^16^, ^17^, ^18^, ^19^ ^]^ generative adversarial networks,^[^ ^20^, ^21^, ^22^ ^]^ variational autoencoders (VAEs),^[^ ^23^, ^24^, ^25^, ^26^, ^27^ ^]^ diffusion models,^[^ ^28^, ^29^ ^]^ normalizing flows,^[^ ^30^ ^]^ and large language models.^[^ ^31^ ^]^ The trained ML models can be further used for high‐throughput screening or conditioned upon physical properties to achieve the inverse design of polymers from properties of interest, such as glass transition temperature (*T*_g_),^[^ ^17^, ^18^ ^]^ thermal conductivity,^[^ ^19^, ^32^, ^33^ ^]^ bandgap^[^ ^26^ ^]^ and gas‐separation properties.^[^ ^19^, ^34^ ^]^ The success of these ML models depends on the choice of suitable representations, which is challenging due to the discrete and undefined degrees of freedom of molecules and polymers. To date, researchers have employed strings,^[^ ^35^, ^36^ ^]^ molecular fingerprints,^[^ ^37^ ^]^ and graphs^[^ ^38^ ^]^ to represent molecules and monomers in ML models. Recently, Yan et al.^[^ ^39^ ^]^ have utilized a pretrained VAE with an artificial neural network for property prediction and virtual screening of vitrimers. While they have discovered a vitrimer with desirable properties, the design space is limited to 184 commercially available monomers, as their VAE is only used as a representation method instead of a generative model. In contrast to virtual screening by forward predictive models, which requires a predefined pool of candidates to screen, generative models are able to learn the distribution of the training data and facilitate the discovery and design of novel chemistries. In the context of using generative models for the inverse design of polymers, previous studies^[^ ^20^, ^21^, ^22^, ^23^, ^24^, ^25^, ^26^, ^27^, ^28^, ^29^, ^30^, ^31^ ^]^ have primarily focused on the design of single molecules or monomers, without addressing the challenges involved in designing multi‐component polymers. In this work, we propose a graph VAE model employing dual graph encoders and overlapping latent dimensions^[^ ^40^, ^41^ ^]^ which enable representation of multi‐component vitrimers and controlled design of selective components simultaneously.

Last few years have seen an increased contribution to structures and properties databases of polymers and molecules such as ZINC15,^[^ ^42^ ^]^ ChemSpider,^[^ ^43^ ^]^ and PubChem.^[^ ^44^ ^]^ However, a dataset of vitrimers to train such a deep generative model is lacking. Furthermore, part of the dataset needs to be associated with the property of interest to enable property‐guided inverse design. Vitrimers are characterized by two key thermal properties: glass transition temperature (*T*_g_) and topology freezing temperature (*T*_v_). *T*_g_ describes the transition from glassy state to rubbery state while *T*_v_ describes the transition from viscoelastic solids to viscoelastic liquids. At service temperatures, vitrimers perform like traditional polymers, but when heated to *T*_v_, the chains gain mobility and carry out exchange reactions at the reactive sites. Traditionally, vitrimer polymers exhibit *T*_v_ > *T*_g_^[^ ^11^, ^45^ ^]^; this makes their future application easier since *T*_g_ dictates design protocols, and healing in vitrimers happens at temperatures higher than *T*_g_. Therefore, in this work, we focus our efforts on designing vitrimers with targeted *T*_g_. We build the first vitrimer dataset derived from the online database ZINC15^[^ ^42^ ^]^ and calculate *T*_g_ by calibrated MD simulations on a subset of vitrimers (Figure 1a,b).

Leveraging this vitrimer dataset, we build an integrated MD‐ML framework for discovery of bifunctional transesterification vitrimers with desirable properties specifically targeted *T*_g_ for the scope of this work (Figure 1b). Each vitrimer contains two reactive constituents (i.e., carboxylic acid and epoxide) with a 1:1 molar ratio which is the predominant ratio used to synthesize transesterification vitrimers in previous studies.^[^ ^3^, ^46^, ^47^, ^48^, ^49^, ^50^ ^]^ Furthermore, the discrete nature of the molecules prohibits a smooth and continuous design space. For example, while molecules are interpretable to human, they are not interpretable to a numerical optimizer for design of vitrimers. To this end, we develop a VAE that receives as input a vitrimer represented by graphs and subgraphs of the constituents and produces a smooth and continuous latent space. In such a latent space, two similar vitrimers are located close to each other while an optimizer can traverse the space of all possible vitrimers. Our unique VAE framework offers both constituent‐specific and joint latent spaces of the chemical constituents, i.e., continuous screening and optimization can be performed on just one or both of the constituents. This enables interpretability on the effects of optimizing over, e.g., acid only, epoxide only, or simultaneously acid and epoxide molecules. The efficacy of the framework is demonstrated by discovering novel vitrimers with *T*_g_ both within and well beyond the dataset. Specifically, while the *T*_g_ in the training data ranges from 250 to 500 K, we discover vitrimers with *T*_g_ ≈569 and 248 K (Figure 1c). To validate the framework predictions, we generate vitrimer chemistries with a *T*_g_ of 323 K (50 °C). We chose this *T*_g_ range to develop vitrimers comparable to commonly used polyamides that find applications in transportation, electronics, consumer goods, and packaging. We examine the top candidates suggested by framework and use chemical intuition to further ensure simpler synthesizability and thermodynamic stability (Figure 1d). The novel, synthesized vitrimer is characterized and experimental *T*_g_ is in good agreement with inverse design target *T*_g_. This validates the proposed framework, which is sufficiently general to be applied to different types of vitrimers and their properties, as a tool for polymer chemists to discover and synthesize novel vitrimer chemistries with desirable properties.

## 2 Results

### 2.1 Design Space and Data Generation

**Figure 2:**
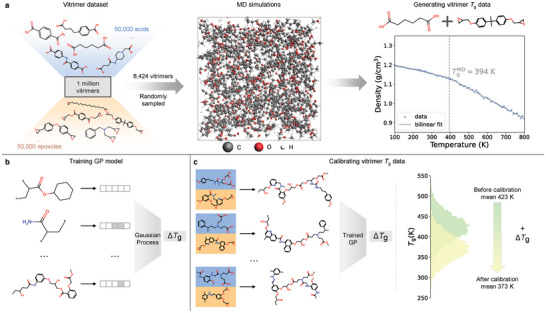
Data generation by MD simulations and calibration by GP model. a) The vitrimer dataset is obtained by randomly sampling one million combinations between 50 000 bifunctional carboxylic acids and 50 000 epoxides derived from the ZINC15 database. MD simulations are carried out to calculate *T*_g_ on a subset of 8424 vitrimers. b) We train a GP model to predict experiment‐MD offset Δ*T*_g_ with a training set of 295 polymers with experimental *T*_g_ in literature. c) Using the trained GP model, we calibrate MD‐calculated *T*_g_ of the vitrimer dataset. The calibrated *T*_g_, serving as a proxy of experimental *T*_g_, is the design target of this work.

We begin by creating a vitrimer dataset to train the VAE model. Since there are only a few available bifunctional transesterification vitrimers recorded in literature, we create a dataset of hypothetical vitrimers by combining carboxylic acids and epoxides. We first build two datasets by collecting available bifunctional carboxylic acids and epoxides from the online chemical compound database ZINC15.^[^ ^42^ ^]^ To further broaden the chemical space, we augment the datasets by adding hypothetical carboxylic acids and epoxides derived from available alcohols, olefins and phenols in the ZINC15 database. In both datasets, molecules satisfying all the following rules are kept: i) Carboxylic acid and epoxide‐containing monomers have exactly two occurrences of their defining functional group (to restrict compositions to linear chains). ii) Molecules with molecular weight smaller than 500 g mol^−1^ (to restrict the sizes of the molecular graphs and facilitate training of the graph VAE). iii) Molecules with C, H, N, O elements only (to emulate the existing transesterification vitrimers). After filtering, two datasets of ≈322 000 carboxylic acids and ≈625 000 epoxides are constructed. To ensure synthesizability, we select the 50 000 acids and 50 000 epoxides with lowest synthetic accessibility (SA) scores^[^ ^51^ ^]^ (i.e., those predicted to be easiest to synthesize). The final dataset is built by randomly sampling one million vitrimers from the design space of 2.5 billion possible combinations between the selected acids and epoxides, as shown in **Figure** **2**a.

To achieve property‐guided inverse design, we further compute *T*_g_ of the vitrimers. Since MD simulations of the entire one‐million dataset are computationally intractable, we calculate *T*_g_ of 8424 vitrimers randomly sampled from the dataset. The quantity can cover a sufficient amount of vitrimers in the design space as well as keep the computational cost to a reasonable level. For each vitrimer, we create a virtual specimen then minimize and anneal the structure to remove local heterogeneities by slowly heating it to 800 K. A snapshot of the annealed system of an example vitrimer (adipic acid and bisphenol A diglycidyl ether) is shown in Figure 2a. The annealed structure is held at 800 K for an additional 50 ps and five specimens separated by 10 ps are obtained. To measure densities at different temperatures for *T*_g_ calculation, each specimen is cooled down from 800 to 100 K linearly in steps of 10 K. By fitting a bilinear regression to the density‐temperature profile, we calculate *T*_g_ as the intersection point of the two linear regressions (Figure 2a). Five replicate simulations are carried out from each specimen to reduce the noise due to the stochastic nature of MD. The distributions of average *T*_g_ and coefficient of variation (i.e., ratio of the standard deviation to the mean) in *T*_g_ of the vitrimers calculated by the five replicate MD simulations are shown in Figure S2a,b (Supporting Information), respectively. The coefficients of variation in *T*_g_ of most of the vitrimers are below 0.1 with only a few ≈0.15, indicating the low uncertainty in our MD simulations. More details on MD simulations are provided in Supporting Information.

Due to the large difference in the cooling rate between MD simulations and experiments, MD‐calculated *T*_g_ is typically overestimated compared with experimental measurements. Compensating for this artifact, Afzal et al. have achieved good correlation between MD‐simulated *T*_g_ and experimental *T*_g_ on 315 polymers using ordinary least squares.^[^ ^52^ ^]^ However, we find empirically that a simple two‐parameter linear fit is insufficient to reduce the effect of larger noise in our MD simulations with smaller systems and fewer replicates (Figure S3c, Supporting Information). Instead, we employ a Gaussian process (GP) regression model to calibrate MD calculations against available experimental data. GP is a probabilistic model that uses a kernel (covariance) function to make probabilistic predictions based on the distance between the queried data point and a training set.^[^ ^53^ ^]^ In order to construct a training dataset for the GP model, we gather 292 polymers from the Bicerano Handbook^[^ ^54^ ^]^ and the Chemical Retrieval on the Web (CROW) polymer database,^[^ ^55^ ^]^ each with documented experimental *T*_g_. If the same polymer appears in both literature sources with different recorded *T*_g_, the average of both values is calculated as final *T*_g_. The available experimental data of three bifunctional transesterification vitrimers^[^ ^11^, ^49^, ^56^ ^]^ is also included and the dataset contains 295 polymers in total. The selected polymers cover a diverse chemical space with a wide *T*_g_ range (171–600 K) which are suitable to calibrate the MD *T*_g_ of designed vitrimers. Additionally, the dataset includes fully cured thermosets and vitrimers whose recorded experimental *T*_g_ values are obtained from the literature with potentially minor variations in crosslinking. These variations are accounted for by the calibration process that uses the experimental and MD simulation data. We compute *T*_g_ for this experimental polymer dataset using the MD protocols described above and calculate the experiment‐MD offset Δ*T*_g_ for each of these polymers. To numerically represent both the polymers within the training set and the vitrimers to be calibrated as inputs for the GP model, we apply extended‐connectivity fingerprints (ECFPs)^[^ ^37^ ^]^ to the repeating units of the polymers, where asterisks (*) indicate connection points. We train the GP model to predict Δ*T*_g_ from molecular fingerprints (see Figure 2b), i.e.,

(1) $$\Delta T_{g}^{\mathrm{GP}}=\mathrm{GP}\left(x\right)$$

where $\Delta T_{g}^{\mathrm{GP}}$ is the predicted experiment‐MD *T*_g_ offset by the GP model and x is the molecular fingerprint of the polymer repeating unit. The calibrated *T*_g_ is defined as

(2) $$T_{g}=T_{g}^{\mathrm{MD}}+\Delta T_{g}^{\mathrm{GP}}$$

where $T_{g}^{\mathrm{MD}}$ is the MD‐calculated *T*_g_. More details on molecular fingerprints and the kernel function are provided in Supporting Information.

To evaluate the performance of our GP model, we implement leave‐one‐out cross validation (LOOCV). In this process, we train our GP model on all data points in the training set except one point and predict its calibrated *T*_g_. Therefore, the one point that is left out serves as a test set and assesses the capabilities of the GP model to predict Δ*T*_g_ of unseen data. We repeat this process for all 295 polymers in the training set and compare the calibrated *T*_g_ (derived from Equation 2) with experimental *T*_g_ recorded in literature. The mean absolute error (MAE) is 28.07 K (Figure S3, Supporting Information), which is comparable to the results reported by Afzal et al.^[^ ^52^ ^]^ (MAE = 27.35 K) with larger systems and longer simulation time. The error is partially attributed to inconsistencies in recorded experimental *T*_g_ between the two literature sources.^[^ ^54^, ^55^ ^]^ Experimentally measured *T*_g_ values for some polymers (such as polypropylene, polyvinylcyclohexane, etc.) differ by ≈30 K gathered from two literature sources.^[^ ^54^, ^55^ ^]^ By utilizing the comprehensive GP model trained on the entire training dataset without LOOCV, we proceed to calibrate *T*_g_ of the vitrimer dataset calculated by MD simulations. The distributions of *T*_g_ of the vitrimers before and after GP calibration are shown in Figure 2c and both distributions approach Gaussian. The average *T*_g_ before and after calibration is 423 and 373 K, respectively. Since the cooling rate in our MD simulations is 12 orders of magnitude higher than typical experiments, the offset of 50 K is consistent with the Williams–Landel–Ferry theory that estimates an increase in *T*_g_ of 3 to 5 K per order of magnitude increase in the cooling rate.^[^ ^57^ ^]^ Ten vitrimers with highest and lowest calibrated *T*_g_ are shown in Figure S4 (Supporting Information), indicating the wide chemical and property space covered by the dataset. In this work, we denote *T*_g_ as the calibrated value from MD simulations, which serves as a proxy of the true experimental *T*_g_. It is also the input to the variational autoencoder and target of inverse design.

### 2.2 Variational Autoencoder

The discrete nature of molecules makes it challenging for the generative model to learn a continuous latent space from discrete data of vitrimers. Any two molecules can have different degrees of freedom (e.g., number of atoms and bonds) and extra attention needs to be paid to the choice of representations. Here we adopt the hierarchical graph representation of molecules developed by Jin et al.^[^ ^25^ ^]^ A molecule is first represented as a graph G=(V,E) with atoms as nodes V and bonds as edges E. We decompose the molecule G into *n* motifs $M_{1},\text{…},M_{n}$. Each motif $M_{i}=\left(V_{i},E_{i}\right)$ where i∈{1,…,n} is a subgraph with atoms $V_{i}$ and edges $E_{i}$. The ensuing step involves a three‐level hierarchical graph representation (schematic illustration in Figure S5, Supporting Information). The motif level $G_{M}$ establishes macroscopic connections in a tree‐like structure, the attachment level $G_{A}$ encodes inter‐motif connectivity via shared atoms, and the atom level G captures finer atomic relationships. More details about the hierarchical graph representation are presented in Supporting Information.

**Figure 3:**
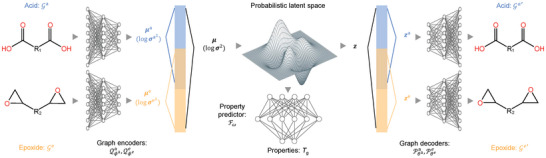
Illustration of the VAE model. The encoders convert acid and epoxide molecules into latent vectors z in a continuous latent space. The latent vectors z are further decoded into acid and epoxide molecules by the decoders. A property predictor is added to predict *T*_g_ from z.

We use a variational autoencoder (VAE) comprising two pairs of hierarchical encoders and decoders associated with the hierarchical representations of acid and epoxide molecules, respectively. A schematic of the framework is presented in **Figure** **3**. Each of the hierarchical encoder uses three message passing networks (MPNs) to encode the graphs from each of the three levels. The acid encoder $Q_{\phi^{a}}^{a}$ (with trainable parameters ϕ^a^) maps the molecular graph of the acid molecule $G^{a}$ into a pair of vectors $\mu^{a}\in R^{d_{a}}$ and $\log{\sigma^{a}}^{2}\in R^{d_{a}}$ of dimension *d*_a_, which are the mean and logarithm variance of a Gaussian distribution. Similarly, $\mu^{e}\in R^{d_{e}}$ and $\log{\sigma^{e}}^{2}\in R^{d_{e}}$ of dimension *d*_e_ are converted from the epoxide molecule $G^{e}$ by the epoxide encoder $Q_{\phi^{e}}^{e}$.

We employ the attributed network embedding method^[^ ^40^, ^41^ ^]^ to obtain the unified mean μ and log variance $\log\sigma^{2}$ of dimension *d* embedding information of the acid and epoxide as well as their unified effects as follows. We define *d*_ae_ = *d*_a_ + *d*_e_ − *d* denoting the overlapping dimensions of $\mu^{a}$ and $\mu^{e}$ and calculate μ by

(3) $$\mu =\underset{\text{acid-specific}}{\underset{︸}{\left[\begin{array}{c} \mu_{1}^{a}\\ \vdots \\ \mu_{d_{a}-d_{\mathrm{ae}}}^{a} \end{array}\right]}}⊕\underset{\text{shared}}{\underset{︸}{\frac{1}{2}\left(\left[\begin{array}{c} \mu_{d_{a}-d_{\mathrm{ae}}+1}^{a}\\ \vdots \\ \mu_{d_{a}}^{a} \end{array}\right]+\left[\begin{array}{c} \mu_{1}^{e}\\ \vdots \\ \mu_{d_{\mathrm{ae}}}^{e} \end{array}\right]\right)}}⊕\underset{\text{epoxide-specific}}{\underset{︸}{\left[\begin{array}{c} \mu_{d_{\mathrm{ae}+1}}^{e}\\ \vdots \\ \mu_{d_{e}}^{e} \end{array}\right]}}$$

where ⊕ denotes vector concatenation. The unified log variance vector $\log\sigma^{2}$ is obtained similarly. Partially overlapping latent dimensions enables both independent and joint control as well as interpretability of embeddings of acid and epoxide. When exploring the latent space and optimizing latent vectors for new vitrimers later, it also allows us to change one part of the vitrimer but keep the other one unaltered.

The unified mean μ and variance $\sigma^{2}$ together describe a diagonal multivariate Gaussian distribution

(4) $$z∼N\left({\left[\begin{array}{c} \mu_{1},\text{…},\mu_{d} \end{array}\right]}^{⊺},\mathrm{diag}\left({\left[\begin{array}{c} \sigma_{1}^{2},\text{…},\sigma_{d}^{2} \end{array}\right]}^{⊺}\right)\right)$$

where z is the latent vector (representation) of dimension *d* encoding necessary information of both input graphs $G^{a}$ and $G^{e}$. To keep differentiability and facilitate the training of VAE, the reparameterization trick^[^ ^58^ ^]^ is used to sample the latent vector z from μ and $\sigma^{2}$ by

(5) $$z=\mu +\varepsilon ⊙{\left[\begin{array}{c} \sigma_{1}^{2},\text{…},\sigma_{d}^{2} \end{array}\right]}^{⊺}$$

where ε∼N(0,I) is a vector of dimension *d* and ⊙ denotes element‐wise multiplication. The acid decoder $P_{\theta^{a}}^{a}$ (with trainable parameters θ^a^) is used to output the acid molecule ${G^{a}}^{\prime }$ from the acid‐specific and shared dimensions of z. Similarly, the epoxide decoder $P_{\theta^{e}}^{e}$ is used to output the epoxide molecule ${G^{e}}^{\prime }$ from the epoxide‐specific and shared dimensions of z. More specifically, the decoders iteratively expand the graphs at three hierarchical levels. At step *t*, three multilayer perceptrons (MLPs) are used to predict the probability distributions of each motif node $p_{M_{t}}^{\prime }$, attachment node $p_{A_{t}}^{\prime }$ and atoms to be attached $p_{{\left(u,v\right)}_{t}}^{\prime }$ (see Supporting Information for more details). An additional MLP is used to predict the probability of backtracing $p_{b_{t}}^{\prime }$, i.e., when there will be no new neighbors to add to the motif node. Both decoders are optimized to accurately reconstruct the molecules, i.e., ${G^{a}}^{\prime }\approx G^{a}$ and ${G^{e}}^{\prime }\approx G^{e}$. Practically this is achieved by minimizing the error between all four predicted probability distributions with respect to the one‐hot encoded ground truth, i.e., $p_{M_{t}}$, $p_{A_{t}}$, $p_{{\left(u,v\right)}_{t}}$ and $p_{b_{t}}$ for *t* = 1,…,*t*_max_ where *t*_max_ is the maximum number of iterations based on depth‐first search of the input molecule (here for simplicity we omit superscripts a and e denoting acid and epoxide). Encoding input vitrimers as described here introduces an information bottleneck^[^ ^59^ ^]^ within the latent representation. This bottleneck selectively retains necessary information required for accurate vitrimer reconstruction while largely reducing the dimensionality and complexity of original data. As a result, vitrimers with similar compositions occupy proximate positions in the latent space.

In order to achieve data‐driven design and uncover novel vitrimers with the interested property, we establish a connection between the latent space and *T*_g_. This is accomplished by employing a neural network surrogate model that takes the latent vectors as inputs and outputs *T*_g_. Consequently, we modify the original VAE architecture and establish a projection from the latent space to *T*_g_ by incorporating the latent vectors z into a property prediction model $F_{\omega }$ (with trainable parameters ω). Thereby, the predicted property is

(6) $$T_{g}^{\prime }=F_{\omega }\left(z\right)$$

We collect two subsets from the vitrimer dataset, one with *N* vitrimers lacking property labels $D=\{\left({G^{a}}^{\left(i\right)},{G^{e}}^{\left(i\right)}\right):i=1,\text{…},N\}$, and one with *N*_prop_ vitrimer and *T*_g_ pairs $D_{\mathrm{prop}}=\left\{\left({G^{a}}^{\left(i\right)},{G^{e}}^{\left(i\right)},T_{g}^{\left(i\right)}\right):i=1,\text{…},N_{\mathrm{prop}}\right\}$. Due to the large difference between *N* and *N*_prop_ (999 000 vs. 7424), we first train the VAE on an unsupervised basis with D and the property predictor is not optimized. Specifically,

(7) $$\begin{array}{cc} \theta^{a},\theta^{e},\phi^{a},\phi^{e}\leftarrow {\text{arg}\;\min}_{\theta^{a},\theta^{e},\phi^{a},\phi^{e}} & \underset{\text{reconstruction loss}}{\underset{︸}{\sum_{i=1}^{N}\left(\mathrm{CE}\left({p_{M}^{\left(i\right)}}^{\prime },p_{M}^{\left(i\right)}\right)+\mathrm{CE}\left({p_{A}^{\left(i\right)}}^{\prime },p_{A}^{\left(i\right)}\right)+\mathrm{CE}\left({p_{\left(u,v\right)}^{\left(i\right)}}^{\prime },p_{\left(u,v\right)}^{\left(i\right)}\right)+\mathrm{BCE}\left({p_{b}^{\left(i\right)}}^{\prime },p_{b}^{\left(i\right)}\right)\right)}}\\ + & \underset{\text{Kullback--Leibler divergence}}{\underset{︸}{\lambda_{\mathrm{KL}}\frac{1}{N}\sum_{i=1}^{N}D_{\mathrm{KL}}\left(N\left({\left[\begin{array}{c} \mu_{1}^{\left(i\right)},\text{…},\mu_{d}^{\left(i\right)} \end{array}\right]}^{⊺},\mathrm{diag}\left({\left[\begin{array}{c} \sigma_{1}^{{\left(i\right)}^{2}},\text{…},{\sigma_{d}^{\left(i\right)}}^{2} \end{array}\right]}^{⊺}\right)\right)∥\;N\left(0,I\right)\right)}} \end{array}$$

where CE and BCE denote cross entropy loss and binary cross entropy loss,^[^ ^60^ ^]^ respectively. For simplicity, the subscript *t* and superscripts a and e are omitted and all terms in reconstruction loss represent the sum over all decoding steps and over acid and epoxide. λ_KL_ > 0 is the regularization weight for Kullback–Leibler divergence. Training the VAE with D aims to construct well‐trained encoders and decoders capable of accommodating a diverse array of vitrimers. The reconstruction loss ensures the accurate reconstruction of the encoded vitrimers with respect to both acid and epoxide molecules by the VAE. The Kullback–Leibler divergence (KLD)^[^ ^61^ ^]^ is a statistical measure to quantify how different two distributions are from each other. Hence, by employing it as a loss term,^[^ ^58^ ^]^ we minimize the difference between the probability distribution of the latent space created by the encoder and the standard Gaussian distribution N(0,I). This helps in constructing a seamless and continuous latent space from which new samples can be generated using standard Gaussian distribution and allows us to discover and design novel vitrimers not present in the training set. The KLD is calculated as

(8) $$\begin{array}{ccc} & & D_{\mathrm{KL}}\left(N\left({\left[\begin{array}{c} \mu_{1},\text{…},\mu_{d} \end{array}\right]}^{⊺},\mathrm{diag}\left({\left[\begin{array}{c} \sigma_{1}^{2},\text{…},\sigma_{d}^{2} \end{array}\right]}^{⊺}\right)\right)∥\;N\left(0,I\right)\right)\\ & & \;=\frac{1}{2}\sum_{j=1}^{d}\left[\mu_{j}^{2}+\sigma_{j}^{2}-\log\left(\sigma_{j}^{2}\right)-1\right] \end{array}$$

Subsequently, we use $D_{\mathrm{prop}}$ to jointly train encoder, decoder and property predictor at the same time, i.e.,

(9) $$\begin{array}{cc} \theta^{a},\theta^{e},\phi^{a},\phi^{e},\omega \leftarrow \text{arg}\;\min_{\theta^{a},\theta^{e},\phi^{a},\phi^{e},\omega } & \underset{\text{reconstruction loss}}{\underset{︸}{\sum_{i=1}^{N_{\mathrm{prop}}}\left(\mathrm{CE}\left({p_{M}^{\left(i\right)}}^{\prime },p_{M}^{\left(i\right)}\right)+\mathrm{CE}\left({p_{A}^{\left(i\right)}}^{\prime },p_{A}^{\left(i\right)}\right)+\mathrm{CE}\left({p_{\left(u,v\right)}^{\left(i\right)}}^{\prime },p_{\left(u,v\right)}^{\left(i\right)}\right)+\mathrm{BCE}\left({p_{b}^{\left(i\right)}}^{\prime },p_{b}^{\left(i\right)}\right)\right)}}\\ + & \underset{\text{Kullback--Leibler divergence}}{\underset{︸}{\lambda_{\mathrm{KL}}\frac{1}{N_{\mathrm{prop}}}\sum_{i=1}^{N_{\mathrm{prop}}}D_{\mathrm{KL}}\left(N\left({\left[\begin{array}{c} \mu_{1}^{\left(i\right)},\text{…},\mu_{d}^{\left(i\right)} \end{array}\right]}^{⊺},\mathrm{diag}\left({\left[\begin{array}{c} \sigma_{1}^{{\left(i\right)}^{2}},\text{…},{\sigma_{d}^{\left(i\right)}}^{2} \end{array}\right]}^{⊺}\right)\right)∥\;N\left(0,I\right)\right)}}\\ + & \underset{\underline{\text{property prediction loss}}}{\underset{︸}{\frac{1}{N_{\mathrm{prop}}}\sum_{i=1}^{N_{\mathrm{prop}}}{\left({T_{g}^{\left(i\right)}}^{\prime }-T_{g}^{\left(i\right)}\right)}^{2}}} \end{array}$$

The additional property prediction loss ensures accurate prediction of *T*_g_ from latent vectors. This joint training process reorganizes the latent space and places vitrimers with similar *T*_g_ in close proximity to each other. More details about hierarchical encoder and decoder, network architecture, training protocols and hyperparameters are presented in Supporting Information.

### 2.3 Performance of the VAE

We first evaluate the ability of the VAE to reconstruct a given vitrimer. We encode the vitrimers in the test set into mean vectors of latent distribution μ then decode μ back to vitrimers. The ratio of successfully reconstructed (i.e., both carboxylic acid and epoxide decoded from μ are identical to input molecules) is 89.1%, which demonstrates well‐trained encoders and decoders capable of accommodating and reconstructing vitrimers unseen by the VAE. Examples of ten vitrimers from the test set and the corresponding reconstructions are presented in Figure S6 (Supporting Information). Eight vitrimers are perfectly reconstructed, while one component of vitrimers is decoded into different but similar molecules in the two unsuccessful examples.

We then assess the performance of the VAE to generate vitrimers. We sample 1000 latent vectors z from standard Gaussian distribution and decode them into the carboxylic acid and epoxide molecules constituting vitrimers. 82.9% of the sampled vitrimers are valid, i.e., the composing acid and epoxide molecules are chemically valid and contain exactly two carboxylic acid and epoxide groups. While it is possible to enforce the VAE model to output molecules only with exactly two functional groups, we choose to keep the current model simple without adding extra computational expenses. All randomly sampled latent vectors are decoded into chemically valid molecules and most of them contain the desired functionality, which is sufficient for our inverse design purposes. Examples of sampled vitrimers are shown in Figure S7 (Supporting Information). Components of the three invalid sampled vitrimers are also carboxylic acids and epoxides but do not have exactly two functional groups.

Apart from validity, we are also interested in the novelty and uniqueness of the generated vitrimers, which are defined as the ratio of sampled vitrimers which are not present in the training set and the expected fraction of unique vitrimers per sampled vitrimers, respectively. Results show that all of the 1000 vitrimers sampled from the latent space are novel and unique, which greatly benefits the discovery of vitrimers by exploring the latent space.

We further examine the effect of joint training with the small dataset $D_{\mathrm{prop}}$ containing a limited number of labeled vitrimers. All four metrics of the model before and after joint training are presented in Table S3 (Supporting Information). The improved reconstruction accuracy and sample validity show that the second‐step joint training enhances the performance of the model and that the encoders and decoders are not biased to the limited data in $D_{\mathrm{prop}}$.

The property predictor maps latent space encoded from vitrimers to *T*_g_ and serves as a surrogate model for estimating *T*_g_ without the need for costly MD simulations. We evaluate the predictive power of the property predictor network by encoding the vitrimers in the test set into mean vectors μ and predicting the associated *T*_g_. The predicted *T*_g_ and true *T*_g_ are compared in Figure S8 (Supporting Information). A mean absolute error of 13.53 K indicates accurate prediction of *T*_g_ by the property predictor which facilitates the inverse design process.

The VAE jointly trained with the property predictor organizes the latent space such that vitrimers exhibiting similar properties are positioned in the vicinity of each other. We examine the distribution of latent vectors and corresponding *T*_g_ of the labeled datasets using principal component analysis (PCA). As shown in Figure S9a,b (Supporting Information), the distribution of the latent vectors shows an obvious gradient in both training and test sets, where vitrimers with higher *T*_g_ cluster in the lower left region. Such a well‐structured latent space based on properties benefits the discovery of novel vitrimers with desired *T*_g_. For comparison, the latent vector distribution before joint training is presented in Figure S9c,d (Supporting Information). The much less obvious trend confirms the effect of joint training on latent space organization.

### 2.4 Interpretable Exploration of the Latent Space

**Figure 4:**
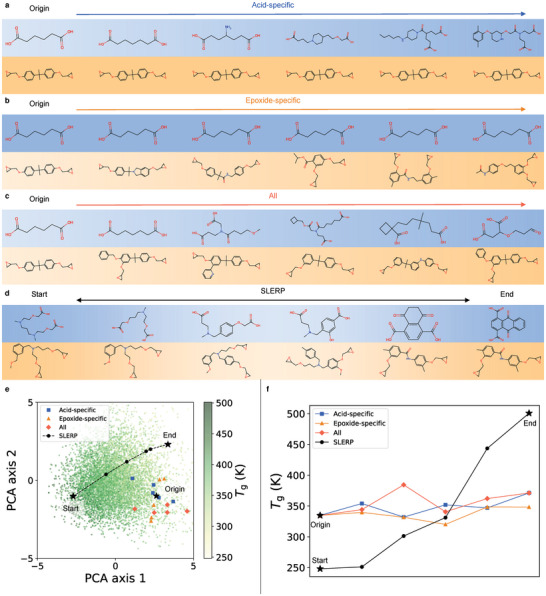
Exploration in the latent space to discover novel vitrimers. Starting with a known vitrimer as origin (adipic acid and bisphenol A diglycidyl ether), vitrimers are discovered by perturbing its latent vector in a) acid‐specific dimensions, b) epoxide‐specific dimensions and c) all dimensions. d) Novel vitrimers are identified along the interpolation path between two vitrimers in the training set. e) The distribution of discovered vitrimers is visualized in the latent space by PCA. f) *T*_g_ of discovered vitrimers. All presented *T*_g_ values are validated by MD simulations and GP calibration.

The well‐trained, continuous latent space enables us to discover new vitrimers by exploring the latent space through modifications of latent vectors z. For example, we start with the latent vector $z_{0}$ of a known vitrimer (adipic acid and bisphenol A diglycidyl ether) as origin and sample latent vectors in the neighborhood by perturbing $z_{0}$. Previous works that employ multi‐component VAEs (i.e., VAEs with multiple encoders and decoders) simply add embedding or mean (log variance) vectors from encoders to derive the unified latent vector z.^[^ ^34^ ^]^ The effect of different components is not considered individually and a change in z leads to potential changes in all components. The partially overlapping latent dimensions (Equation 3) allow us to explore the vicinity of the origin $z_{0}$ along different axes by adding noise to acid‐specific latent dimensions (first *d*_a_ dimensions of $z_{0}$), epoxide‐specific latent dimensions (last *d*_e_ dimensions of $z_{0}$) and all latent dimensions of $z_{0}$ (details are provided in Supporting Information). Consequently, novel vitrimers with changes in only acid, only epoxide and both components are identified by decoding the latent vectors modified along three axes, as shown in **Figures** **4**a–c. The decoded vitrimers present variety in molecular structures without significant changes in *T*_g_ (Figure 4f) due to limited search region in latent space, which opens an opportunity to tailor a specific vitrimer to its novel variants with different molecular structures but preserve certain property similarity.

Besides neighborhood search, we perform an interpolation between two points in the latent space and identify a series of new vitrimers along the path. Figure 4d presents an example of spherical interpolation (SLERP)^[^ ^62^ ^]^ between vitrimers with highest and lowest *T*_g_ in the training set. As opposed to linear interpolation (LERP), we use SLERP because Gaussian distribution in high dimensions closely follows the surface of a hypersphere. The decoded vitrimers show a smooth transition from the low‐*T*_g_ vitrimer with linear structure to the high‐*T*_g_ vitrimer with more aromatic nature. The continuous transition between molecular structures and *T*_g_ (Figure 4f) evidences the smoothness of the latent space with associated *T*_g_. The vitrimers discovered by LERP and their associated *T*_g_ are shown in Figure S10 (Supporting Information). More details on spherical and linear interpolation schemes are presented in Supporting Information.

### 2.5 Inverse Design by Bayesian Optimization

The VAE together with the property predictor succeeds in learning the hidden relationships between latent space and *T*_g_ of vitrimers, which allows us to tailor vitrimer compositions to desirable *T*_g_ even beyond the training regime. Although we have achieved forward projection from the vitrimer space (or latent space) to property space, the inverse mapping is more challenging due to the fact that multiple distinct vitrimers could have a similar *T*_g_. To achieve inverse design of vitrimers with optimal or desirable *T*_g_, we employ batch Bayesian optimization to identify the latent vectors z that have the potential to be decoded into vitrimers with target *T*_g_. The proposed candidates are further validated by MD simulations with GP calibration, and the optimal solutions with desirable *T*_g_ are found. Due to the discrete nature of molecules, the latent vectors proposed by the optimization process may lead to invalid molecules. Furthermore, since the discrete molecules are projected onto a continuous latent space by the VAE, it is inevitable that multiple distinct latent vectors in the neighborhood can be decoded into the same vitrimer but are associated with different *T*_g_ predicted by the property predictor. This severely limits the accuracy and efficiency of the optimization process. To this end, we add an additional decoding‐encoding step before passing z to the property predictor to predict *T*_g_ (Figure S11, Supporting Information). More specifically, when evaluating the *T*_g_ of a point of interest z in the latent space during the optimization process, z is first decoded into a carboxylic acid and an epoxide. If both molecules are valid, they are passed to the encoders to obtain the reconstructed mean vector $\mu_{\mathrm{recon}}$, which is further passed to the property predictor to evaluate the *T*_g_. In this way, the Bayesian optimization algorithm is able to search for potential candidates with desirable *T*_g_ efficiently without proposing the same vitrimer for a large number of iterations. More details about Bayesian optimization are provided in Supporting Information.

**Figure 5:**
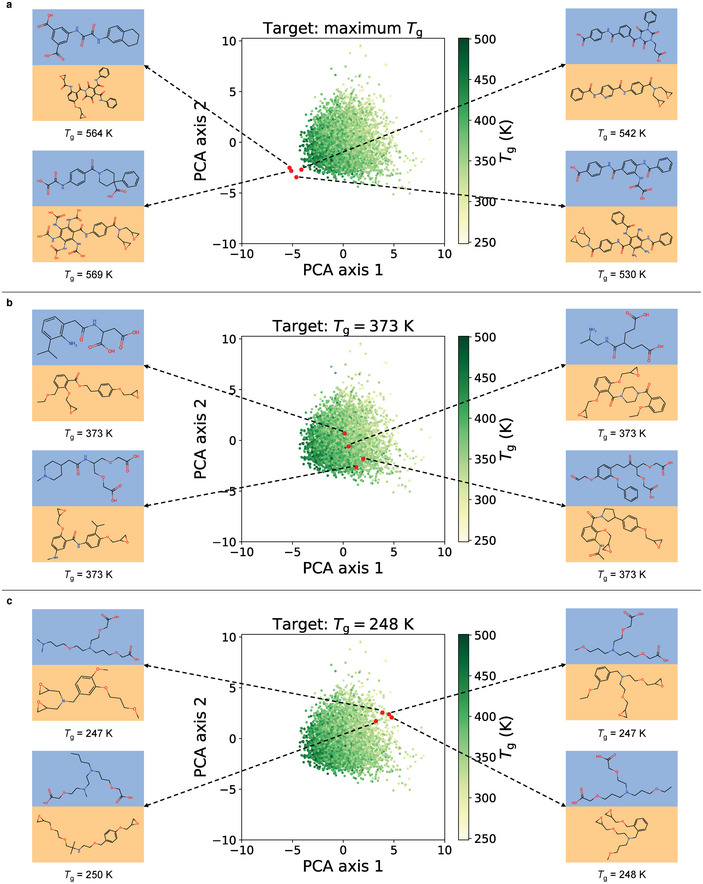
Inverse design of novel vitrimers by Bayesian optimization based on three targets of desirable *T*_g_. a) Maximum *T*_g_, b) *T*_g_ = 373 K and c) *T*_g_ = 248 K. All presented *T*_g_ values of proposed vitrimers are validated by MD simulations and GP calibration.

To demonstrate the effectiveness of our inverse design framework, we use Bayesian optimization to discover novel vitrimers with three different targets: maximum *T*_g_, *T*_g_ = 373 K and *T*_g_ = 248 K. *T*_g_ of the proposed candidates is validated by MD simulations and GP calibration. For each target, four examples of discovered vitrimers are presented in **Figure** **5**. For the first target (maximum *T*_g_), our VAE model generates novel vitrimers with MD‐validated *T*_g_ beyond the upper bound of *T*_g_ in the training data (500 K) and thereby expands the limits in thermal properties of bifunctional transesterification vitrimers. The Bayesian optimization procedures are able to probe the latent space outside of the training domain and propose novel vitrimers with extreme properties, which is difficult for traditional forward modeling methods to find. For the second target, the Bayesian optimization algorithm effectively searches the latent space and successfully proposes vitrimers with the exact target *T*_g_ of 373 K. The corresponding latent vectors are spread out in the latent space and the vitrimer compositions present significant molecular variety. For the third target which is the lower bound of the training domain (248 K), the proposed vitrimers (especially carboxylic acids) are more similar to each other and occupy a small region in the latent space. This can be attributed to the fact that there are not many linear molecules with more aliphatic nature in the 50 000 acids or epoxides making the training set. As a result, the distribution of these vitrimers with low *T*_g_ is insufficiently captured by the VAE and the proposed candidates of low‐*T*_g_ vitrimers are restrained by the limited training data.

The *T*_g_ distributions of the dataset and ≈100 designed vitrimers for each target are presented in Figure S12 (Supporting Information). For the target of maximum *T*_g_, our model efficiently discovers novel vitrimer chemistries beyond the training property space. For the other two targets of 373 K and 248 K, the distributions are centered around the design target. Ten examples of novel vitrimers discovered by Bayesian optimization for each target are presented in Figure S13 (Supporting Information). For the first target, all ten proposed vitrimers have validated *T*_g_ larger than 500 K, which indicates the effective extrapolation beyond the training domain by our framework. For the other two targets of finding vitrimers with exact target *T*_g_, the discovered vitrimers present *T*_g_ within a range of 2 K around the target and maintain considerable molecular diversity, proving the high accuracy in the inverse design process. We further examine the stability of the proposed molecules by minimizing them by reactive molecular dynamics (ReaxFF) using the CHON2017_weak_bb force field.^[^ ^9^ ^]^ All molecules remain stable during minimization and the minimized structures are presented in Figure S14 (Supporting Information). To ensure the feasibility of applying the calibration GP model trained by 295 polymers to the discovered novel vitrimers, in Figure S15 (Supporting Information) we present their molecular fingerprints reduced to two dimensions by PCA. The calibration dataset occupies a broad chemical space and covers the chemistries of designed vitrimers.

We carry out further analysis based on the molecular descriptors of ten proposed vitrimers for each target. The molecular descriptors except density are calculated from the vitrimer repeating units (*n* = 1 in Figure S1a, Supporting Information) by the Modred package.^[^ ^63^ ^]^ Density of each vitrimer at 300 K is extracted from MD simulations. The relevant descriptors of designed low, medium and high‐temperature vitrimers are presented in Figure S16 (Supporting Information). The vitrimers with higher *T*_g_ have larger molecular weight, higher density, more heavy atoms and multiple bonds, and fewer rotatable bonds. Consequently, the chains in these vitrimers are more rigid and less mobile, which agrees with the common knowledge of structure‐*T*_g_ relationships in polymers.

We compare *T*_g_ of the designed vitrimers with nine commonly used polymers in Figure S17 (Supporting Information). The proposed vitrimers cover a wide range of *T*_g_ suitable for various applications from coating materials to aerospace polymers. With further tuning of the target, our framework has the potential to discover vitrimer compositions with any *T*_g_ within an expanded range and expedite the widespread applications of sustainable polymers in various industries.

### 2.6 Experimental Synthesis of Novel Vitrimer Designed with Chemical Intuition

**Figure 6:**
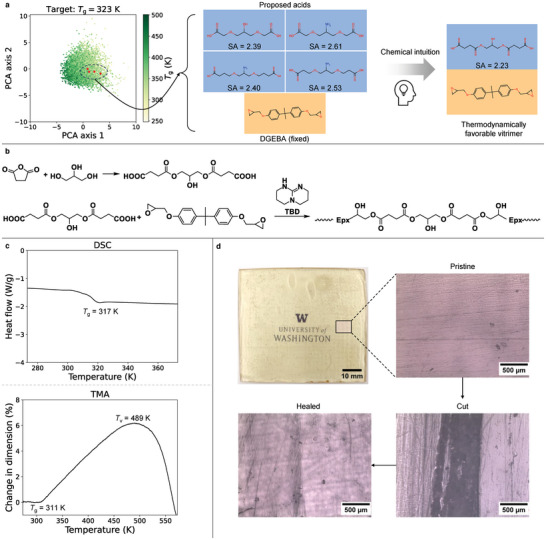
Synthesis and characterization of novel vitrimer designed by ML framework. a) Four vitrimers (epoxide fixed as DGEBA) are proposed by Bayesian optimization with a target *T*_g_ = 323 K. Driven by chemical intuition, a symmetric and thermodynamically stable vitrimer is selected. b) Reaction scheme for synthesis of the acid and consequent crosslinking with DGEBA to form a novel vitrimer. c) Experimental characterization of the synthesized vitrimer to measure *T*_g_ and *T*_v_. The measured *T*_g_ ranges from 311 K to 317 K, which agrees well with the design target. d) Images of pristine, cut and healed vitrimer specimens, confirming healability of the synthesized vitrimer.

To experimentally validate the effectiveness of the VAE model, we perform Bayesian optimization to propose novel vitrimers with a target *T*_g_ of 323 K. Since epoxides are typically more difficult to synthesize, the epoxide molecule is fixed as bisphenol A diglycidyl ether (DGEBA) during optimization to improve the synthesizability of the vitrimer. In other words, the shared and epoxide‐specific dimensions are fixed while we only optimize acid‐specific dimensions in Equation 3. Out of the carboxylic acids proposed by the VAE model, four acids with low SA scores (2.39 to 2.61) and symmetric structures are further analyzed for synthesis feasibility. Additionally, some of these acid molecules have an amine group that can react with epoxide rings to form irreversible covalent bonds, thereby reducing the adaptive nature of the macromolecular network. Keeping these thermodynamical stability issues in polymer synthesis and resulting crosslinked network through chemical intuition, we make slight modifications to the proposed structures to achieve a symmetric acid molecule with a lower SA score (2.23) that can be synthesized using off‐the‐shelf chemicals. This acid is crosslinked with DGEBA epoxide to form a stable polymer, demonstrating inverse design and synthesis of novel vitrimer chemistry (**Figure** **6**a).

The synthesis of the novel vitrimer chemistry is carried out by the ring opening reaction of succinic anhydride by glycerol followed by immediate crosslinking with DGEBA. The reaction of beta‐hydroxy groups of glycerol with succinic anhydride opens the ring and creates carboxylic functional groups. The resultant acid is crosslinked with DGEBA in presence of catalyst triazabicyclodecene (TBD) to yield the final vitrimer product (Figure 6b). The cured vitrimer is verified using Fourier transform infrared (FTIR) spectroscopy, as shown in Figure S18 (Supporting Information). The characteristic peaks for carbonyl groups at 1728 cm^−1^ and for aromatic epoxide chains at 1033 to 1028 cm^−1^ are observed. In addition, other peaks at 1400 to 1608 cm^−1^ indicate the formation of ester linkages between acids and epoxides. We further characterize the thermal properties of the synthesized vitrimer using differential scanning calorimetry (DSC) and thermomechanical analysis (TMA), as shown in Figure 6c. DSC result shows one transition temperature (*T*_g_) at 317 K, indicating complete curing of crosslinked vitrimer. TMA result presents two thermal transitions, first at 311 K for *T*_g_ and second at 489 K for *T*_v_. The difference in *T*_g_ from DSC and TMA arises due to different physical phenomena and sensitivities measured by each technique. DSC measures heat flow associated with the glass transition, which reflects changes in heat capacity as the polymer transitions from a glassy to a rubbery state, it therefore provides an average *T*_g_. TMA on the other hand measures the mechanical response of the polymer to non‐isothermal creep and the machine detects subtle changes in molecular mobility through dimensional changes. Experimental *T*_g_ from DSC and TMA agrees well with the design target (323 K) and demonstrates the capability of our framework to design novel vitrimers with desired experimental *T*_g_. The second transition indicates flowability of the vitrimer at elevated temperature when dynamic exchange reactions start to occur and enhance polymer chain mobility. The mechanical properties of the synthesized vitrimer are measured by tensile testing (Figure S19, Supporting Information), which indicates a tensile stress of 11 MPa and an elastic modulus of 692 MPa (Table S5, Supporting Information). To confirm healability of the vitrimer, we cut a pristine specimen and heal it at temperature around *T*_v_. The surfaces of pristine, cut, and healed samples are examined under the microscope (Figure 6d). The complete removal of damage shows healability of the synthesized novel vitrimer chemistry and the recyclability is validated by the recovered sample using heat press (Figure S20, Supporting Information). To demonstrate the capability of the model in discovering vitrimers with a higher target *T*_g_, another round of Bayesian optimization is conducted to design vitrimers with a target *T*_g_ of 373 K (100 °C). The acid‐specific dimensions are optimized while the epoxide is fixed as DGEBA. The proposed acid molecules include 1,4‐cyclohexanedicarboxylic acid (CHDA) which is a commercially available carboxylic acid. Notably, the vitrimer composed of CHDA and DGEBA with an equal molar ratio has been synthesized and characterized in a previous work^[^ ^64^ ^]^ which reports an experimental *T*_g_ of 358 ± 2.3 K. This value agrees well with our design target and validates the accuracy and reliability of our framework for inverse design of vitrimer chemistries with higher *T*_g_ targets (Figure S21, Supporting Information).

## 3 Conclusion

We develop an integrated MD‐ML framework for inverse design of bifunctional transesterification vitrimers with desirable *T*_g_. A diverse vitrimer dataset is built for the first time from the ZINC15 database.^[^ ^42^ ^]^ High‐throughput MD simulations with a GP calibration model are employed to calculate *T*_g_ on a subset of vitrimers. The dataset is used to train a VAE model with dual graph encoders and decoders which enables representation and design of the desired vitrimer components. This further provides flexibility by exploring the latent space and optimizing latent vectors of different components for novel vitrimers. We demonstrate the high accuracy and efficiency of our framework in discovering novel vitrimers with three different targets of *T*_g_ even beyond the training distribution. The proposed vitrimers achieve both molecular variety and desirable *T*_g_ within 2 K range around the target, which make them ideal candidates for sustainable polymers for different applications. To validate our framework in experiments, we synthesize and characterize a novel vitrimer designed by the model. This vitrimer is proposed by optimizing acid‐specific dimensions of latent vector while fixing epoxide as DGEBA. Driven by chemical intuition, we then slightly modify the proposed acids to a thermodynamically favorable derivative. The experimentally measured *T*_g_ (311 to 317 K) agrees well with the design target (323 K), which validates effectiveness of the VAE model.

While this work focuses on transesterification vitrimers that rely on catalysts, our VAE model can be modified to design catalyst‐free vitrimers composed of anhydrides, epoxides, and co‐curing agents such as glycerol,^[^ ^65^ ^]^ triethanolamine,^[^ ^66^, ^67^ ^]^ and phosphaphenanthrene‐derived diols.^[^ ^68^ ^]^ By incorporating an additional pair of encoder and decoder to embed the necessary information of the co‐curing agent and employing partially overlapping latent dimensions, we can achieve the selective design of any of the three components. This approach is particularly advantageous when optimizing the properties of these vitrimers while preserving specific functionalities. Apart from transesterification vitrimers, our VAE model can be further applied to the design of other vitrimer types, such as disulfide bond exchange and Schiff base vitrimers. The multiple encoder‐decoder pairs and overlapping latent dimension scheme allow for the design of more complex vitrimer systems. In addition, our MD‐ML framework can be potentially extended to a wide range of properties and other types of polymers. Recent advancements in high‐throughput MD simulations have led to the creation of polymer datasets with diverse properties including thermal conductivity,^[^ ^69^, ^70^ ^]^ free volume^[^ ^71^ ^]^ and ionic conductivity.^[^ ^72^ ^]^ With sufficient polymer data from MD simulations, our VAE architecture can be easily adjusted to other types of multi‐component polymers, such as copolymers, thermosets, and covalent organic frameworks. By adjusting the output dimensions of the property predictor, the model can also be extended to multi‐objective inverse design through Bayesian optimization. This enables the definition of Pareto fronts to effectively balance trade‐offs between various properties of interest. The complete workflow of computational design and experimental validation opens an opportunity for polymer scientists to achieve high‐fidelity inverse design of multi‐component polymeric materials with desirable properties.

## 4 Experimental Section

Details of MD simulations (Section S1, Supporting Information), GP calibration model (Section S2, Supporting Information), hierarchical representation of molecules (Section S3.1, Supporting Information), VAE architecture and training protocols (Section S3.2, Supporting Information), VAE performance (Section S3.3, Supporting Information), exploration of the latent space (Section S3.4, Supporting Information), Bayesian optimization (Section S3.5, Supporting Information), estimate of computational efficiency (Section S4, Supporting Information) and experiments (Section S5, Supporting Information) are provided in Supporting Information.

## Conflict of Interest

J.A.S., Z.L., S.Z. and B.H.N. are employees of Microsoft Corporation. Y.Z., P.T., A.K.B., S.K. and A.V. declare no competing interests.

## Author Contributions

Y.Z. contributed to methodology, software, validation, data curation, visualization, writing, and reviewing and editing; P.T. worked on methodology, software, visualization, reviewing and editing; A.K.B. contributed to methodology, experimentation, reviewing and editing; J.A.S. was responsible for conceptualization, methodology, software, and reviewing and editing; Z.L. contributed to software and reviewing and editing; S.Z. participated in reviewing and editing; B.H.N. contributed to conceptualization, reviewing and editing, and supervision; S.K. was responsible for conceptualization, methodology, reviewing and editing, and supervision; and A.V. contributed to conceptualization, methodology, reviewing and editing, and supervision.

## Supporting information

Supporting Information

## Data Availability

The data and code that support the findings of this study are openly available at https://github.com/vashisth‐lab/VitrimerVAE.
