# Supplementary material for: AI‐Guided Inverse Design and Discovery of Recyclable Vitrimeric Polymers
Source: Adv Sci (Weinh). 2024 Dec 16;12(6):2411385. doi: 10.1002/advs.202411385 (PMC11809429; doi:10.1002/advs.202411385)
Supplement: Supplementary file 1 — Supporting Information [file ADVS-12-2411385-s001.pdf]

ADVANCED SCIENCE
Open Access

Supporting Information

for *Adv. Sci.*, DOI 10.1002/advs.202411385

AI-Guided Inverse Design and Discovery of Recyclable Vitrimeric Polymers

Yiwen Zheng, Prakash Thakolkaran, Agni K. Biswal, Jake A. Smith, Ziheng Lu, Shuxin Zheng, Bichlien H. Nguyen, Siddhant Kumar* and Aniruddh Vashisth**

AI-Guided Inverse Design and Discovery of Recyclable Vitrimeric Polymers

Supporting Information

Yiwen Zheng[1] *Prakash Thakolkaran*[2] *Agni K. Biswal*[1] *Jake A. Smith*[3,4] *Ziheng Lu*[5] *Shuxin Zheng*[5] *Bichlien H. Nguyen*[3,4*] *Siddhant Kumar*[2*] *Aniruddh Vashisth*[1*]

[1]Department of Mechanical Engineering, University of Washington, Seattle, WA 98195, USA
[2]Department of Materials Science and Engineering, Delft University of Technology, 2628 CD Delft, The Netherlands
[3]Microsoft Research, Redmond, WA 98052, USA
[4]Paul G. Allen School of Computer Science and Engineering, University of Washington, Seattle, WA 98195, USA
[5]Microsoft Research Asia, Beijing 100080, China
[*]Email: bnguy@microsoft.com, sid.kumar@tudelft.nl, vashisth@uw.edu

S1 Molecular dynamics simulations

We perform MD simulations on 8,424 vitrimers sampled from the large dataset of one million to calculate their T_g. The simulations are conducted by Large-scale Atomic/Molecular Massively Parallel Simulator (LAMMPS) [1] and Polymer Consistent Force Field (PCFF) [2] to describe the potential energy of atoms. PCFF has been applied to simulate the behavior of various polymeric systems including vitrimers [3, 4]. We build the polymer chains in an alternating copolymer manner. For each vitrimer composition (i.e., one carboxylic acid molecule and one epoxide molecule), we connect the acid molecule and opened epoxide molecule alternately to form one vitrimer chain of around one thousand atoms. The reaction scheme is shown in Figure S1a. Four of these chains are placed in a cubic simulation box with a density of 0.5 g/cm^3 (Figure S1b). The connection and placement of atoms in the simulation box are done by Enhanced Monte Carlo package [5], which creates input structures for MD simulations using the Monte Carlo method with energetically favored orientations.

The initial configuration is minimized using the conjugate gradient method and annealed to remove local heterogeneities. Specifically, the minimized structure is first relaxed under NVT ensemble (300 K) for 50 ps and under NPT ensemble (300 K and 1 atm) for 100 ps. We further heat the virtual specimen from 300 K to 800 K under NPT (1 atm) in 500 ps. The dimension of the simulation box is reduced and the density is taken to a realistic level after annealing. Two snapshots of the virtual specimens of an example vitrimer (adipic acid and bisphenol A diglycidyl ether) before and after annealing are presented in Figure S1b. We hold the annealed system at 800 K for an additional 50 ps to obtain five independent specimens separated by 10 ps, which is proved to be sufficient to eliminate the effect of initial structures and ensure better statistics [6]. For production period, each of these specimens is cooled from 800 K to 100 K in a 10 K step. Each cooling step takes 25 ps under NPT followed by a 25-ps holding at constant temperature, during which the density is calculated as the average from 25 frames. We fit a bilinear regression to the density-temperature profile from 800 K to 100 K and the intersection point is defined as the T_g^MD (Figure 2a in the manuscript). All T_g^MD results from five virtual specimens are averaged to reduce the uncertainty due to the stochastic nature of MD simulations. The distributions of mean MD-calculated T_g^MD and coefficient of variation (i.e., mean divided by standard deviation) from five replicates of 8,424 vitrimers are presented in Figure S2.

S2 Gaussian process model for calibration of T_g

To compensate for the overestimation in T_g^{MD} due to the larger cooling rate compared with experiments, a calibration procedure is required. Afzal et al. [7] calibrate T_g^{MD} against experimental T_g by linear regression. However, we find it insufficient due to the effect of increased uncertainty associated with smaller systems in our simulations (Figure S3c). Instead, we calibrate T_g^{MD} from MD simulations against available experimental data in literature using a Gaussian process (GP) regression model. 2048-bit extended-connectivity fingerprints (ECFPs) [8] with radius 3 are used as input to the GP model to represent the repeating units of polymers. For vitrimers (i.e., combinations of carboxylic acids and epoxides), we obtain the repeating units by the reaction scheme depicted in Figure S1a with $n = 1$. For the GP model, we employ the Tanimoto kernel [9, 10], which relies on the Tanimoto similarity measure extensively used in cheminformatics. Unlike the traditional kernels (e.g., the radial basis function kernel used by Jinich et al. [11]) which are more suitable for continuous spaces (Figure S3b), Tanimoto kernel allows for swift and accurate comparison between molecular fingerprints as bit vectors. The Tanimoto kernel in the GP model is defined as follows:

$$k(\boldsymbol{x}, \boldsymbol{x}') = \frac{\sigma \langle \boldsymbol{x}, \boldsymbol{x}' \rangle}{\|\boldsymbol{x}\|^2 + \|\boldsymbol{x}'\|^2 - \langle \boldsymbol{x}, \boldsymbol{x}' \rangle}, \tag{1}$$

where $\boldsymbol{x}, \boldsymbol{x}'$ are fingerprints of two molecules, σ is the variance of the kernel, $\langle \boldsymbol{x}, \boldsymbol{x}' \rangle$ is the inner product and $\|\boldsymbol{x}\|^2$ is the square of norm. Calibrated and experimental T_g from leave-one-out cross validation (LOOCV) with different calibration methods are compared in Figure S3 and GP calibration with Tanimoto kernel achieves the best accuracy with a mean absolute error of 28.07 K. We proceed to calibrate T_g^{MD} of all 8,424 vitrimers by the trained GP model. Ten vitrimers with highest and lowest calibrated T_g are presented in Figure S4.

S3 Machine learning framework

S3.1 Hierarchical representation of molecules

We follow the hierarchical representation of molecules proposed by Jin et al. [12]. A molecule $\mathcal{G} = (\mathcal{V}, \mathcal{E})$ is preprocessed by decomposing it into n motifs (subgraphs) $\mathcal{M}_1, ..., \mathcal{M}_n$ using the following procedures. First, we identify all bonds between atom pair u and v with more than two connections and either u or v belongs to a ring. We then break all these bonds and convert $\mathcal{G}$ into a series of detached graphs. For each detached graph, if it appears more than a threshold frequency f during decomposition of all molecules in the training set, it is selected as a motif. Otherwise it is further decomposed into single rings and bonds (i.e., one bond with two atoms) which are selected as motifs. In this way, chemical validity is preserved and the union of all motifs covers the entire graph. We preprocess all molecules in the training set by the same procedures and a motif vocabulary $V_\mathcal{M}$ is obtained. We empirically find that the VAE model works better when the motifs are constrained to single rings and bonds, i.e., $f \to \infty$. This can be attributed to the fact that all molecules in the dataset are relatively small and limited to a maximum molecular weight of 500 g/mol. Larger motifs with more than one bonds can be used to accommodate larger molecules if necessary. Two vocabularies with sizes of 124 and 104 motifs are constructed for acid and epoxide molecules, respectively.

We further represent $\mathcal{G}$ as a combination of hierarchical graphs at three levels (see Figure S5 for a schematic illustration of the three-level hierarchical representation). The motif level $\mathcal{G}_\mathcal{M}$ captures how motifs $\mathcal{M}_1, ..., \mathcal{M}_n$ are connected, i.e., $\mathcal{G}_\mathcal{M} = (\mathcal{V}_\mathcal{M}, \mathcal{E}_\mathcal{M})$ with motifs as nodes and bonds connecting motifs as edges. In addition, the attachment level $\mathcal{G}_\mathcal{A}$ represents connection between motifs through shared atoms. Each node $\mathcal{A}_i = (\mathcal{M}_i, \{v_j\})$ at this level defines a connection site of $\mathcal{M}_i$ with $\{v_j\}$ as all possible atoms shared by $\mathcal{S}_i$ and its neighbors. Since the possible connection sites of $\mathcal{M}_i$ are finite, a vocabulary of attachment nodes $V_\mathcal{A}(\mathcal{M}_i)$ that depends on each motif $\mathcal{M}_i$ is constructed. Finally, the atom level $\mathcal{G}$ encodes the graph at the atomic level. Each node is an atom and each edge is a bond of the molecule. This representation

scheme captures necessary information of molecules at three levels with different resolutions and ensures chemical validity. As a result, the associative encoder and decoder can achieve accurate reconstruction and efficient generation of valid vitrimers in the VAE framework.

S3.2 VAE architecture and training protocols

We adopt the hierarchical encoder and decoder associated with the hierarchical graph representation [12]. We use PyTorch [13] to implement and train the model, and RDKit [14] for cheminformatics operations.

S3.2.1 Encoder

The hierarchical encoder encodes a molecule $\mathcal{G}$ (which can be either acid or epoxide; superscripts omitted for simplicity) from the finer level to the coarser level. The encoder contains three message passing networks (MPNs) as detailed by Jin et al. [12]. At the atom level, each node is an atom v and each edge is a bond e_{uv} between atoms u and v. The node features consist of atomic charge and atom type and the edge feature is bond type. All atom and bond types are represented as one-hot encodings. The node and edge features are first converted into embedding vectors before passing to the MPN:

$$\{\boldsymbol{h}_v\} = \mathrm{MPN}\left(\mathcal{G}, \{E(v)\}, \{E(e_{uv})\}\right), \tag{2}$$

where $\{\boldsymbol{h}_v\}$ is the atom-level encoding for each atom v and $E(\cdot)$ denotes embedding vector of $(\cdot)$. At the attachment level, the node feature is a concatenation of its embedding $E(\mathcal{A}_i)$ and the sum of all atom-level encodings of its constituent atoms:

$$\boldsymbol{f}_{\mathcal{A}_i} = \mathrm{MLP}\left(E(\mathcal{A}_i) \oplus \sum_{v\in\mathcal{M}_i} \boldsymbol{h}_v\right). \tag{3}$$

The edge feature between attachment nodes $\mathcal{A}_i$ and $\mathcal{A}_j$ is an embedding of a parameter x_{ij} denoting their parent-child relation based on depth-first search:

$$x_{ij} = \begin{cases} 0, & \text{if } \mathcal{A}_j \text{ is the parent;} \\ k, & \text{if } \mathcal{A}_i \text{ is the } k\text{-th child of } \mathcal{A}_j. \end{cases} \tag{4}$$

The attachment-level encodings are calculated as

$$\{\boldsymbol{h}_{\mathcal{A}_i}\} = \mathrm{MPN}\left(\mathcal{G}_{\mathcal{A}}, \{\boldsymbol{f}_{\mathcal{A}_i}\}, \{E(x_{ij})\}\right). \tag{5}$$

The encoding at the motif level is similar to attachment level with nodes as motifs $\mathcal{M}_i$, i.e.,

$$\boldsymbol{f}_{\mathcal{M}_i} = \mathrm{MLP}\left(E(\mathcal{M}_i) \oplus \boldsymbol{h}_{\mathcal{A}_i}\right). \tag{6}$$

and

$$\{\boldsymbol{h}_{\mathcal{M}_i}\} = \mathrm{MPN}\left(\mathcal{G}_{\mathcal{M}}, \{\boldsymbol{f}_{\mathcal{M}_i}\}, \{E(x_{ij})\}\right). \tag{7}$$

Two linear neural networks are used to output mean vector $\boldsymbol{\mu}$ and log variance vector $\log\boldsymbol{\sigma}^2$ from the encoding of the root motif $\boldsymbol{h}_{\mathcal{M}_1}$. The root motif is the first motif to be generated during decoding.

S3.2.2 Decoder

The decoder attempts to iteratively build the hierarchical graph based on latent vector $\boldsymbol{z}$ of the original molecule. Here for simplicity we refer to $\boldsymbol{z}$ as the concatenation of acid-specific dimensions (or epoxide-specific dimensions) with shared dimensions (see Equation 3 in the manuscript). At t-th step of generation, we denote $\mathcal{M}_k$ as the motif whose neighbor will be generated in the next step. Motifs are generated in a

depth-first order. We use the same hierarchical MPN architecture to encode all the motifs and atoms in the partially generated graph and obtain motif encodings $\boldsymbol{h}_{\mathcal{M}_k}$ and atom encodings $\boldsymbol{h}_v$ for each existing motif and atom. At the motif level, the next motif $\mathcal{M}_t$ to be connected to $\mathcal{M}_k$ is predicted based on the entire motif vocabulary $V_{\mathcal{M}}$:

$$\boldsymbol{p}_{\mathcal{M}_t} = \text{softmax}\left(\text{MLP}\left(\boldsymbol{h}_{\mathcal{M}_k} \oplus \boldsymbol{z}\right)\right). \tag{8}$$

At the attachment level, we predict which attachment $\mathcal{A}_t$ of motif $\mathcal{M}_t$ is used, which is classified over the attachment vocabulary $V_{\mathcal{A}}(\mathcal{M}_t)$:

$$\boldsymbol{p}_{\mathcal{A}_t} = \text{softmax}\left(\text{MLP}\left(\boldsymbol{h}_{\mathcal{M}_k} \oplus \boldsymbol{z}\right)\right). \tag{9}$$

At the atom level, the detailed atomic attachment configuration (u, v) is decided, where u and v are atoms from $\mathcal{A}_k$ and $\mathcal{A}_t$, respectively. The probability of a certain attachment configuration $(u, v)_t$ is calculated as

$$\boldsymbol{p}_{(u,v)_t} = \text{softmax}\left(\text{MLP}\left(\boldsymbol{h}_u \oplus \boldsymbol{h}_v\right) \cdot \boldsymbol{z}\right). \tag{10}$$

An additional MLP is used to predict the probability of backtracing, i.e., there are no new neighbors to be generated for $\mathcal{M}_k$:

$$\boldsymbol{p}_{\text{b}_t} = \text{softmax}\left(\text{MLP}\left(\boldsymbol{h}_{\mathcal{M}_k} \oplus \boldsymbol{z}\right)\right). \tag{11}$$

S3.2.3 Training

The one million vitrimer dataset is divided into two subsets: 999,000 vitrimers without T_g as unlabeled training set $\mathcal{D} = \{(\mathcal{G}^{\text{a}(i)}, \mathcal{G}^{\text{e}(i)}) : i = 1, ..., 999000\}$ and 1,000 vitrimers with T_g as test set $\mathcal{D}_\text{test} = \{(\mathcal{G}^{\text{a}(i)}, \mathcal{G}^{\text{e}(i)}, T_\text{g}^{(i)}) : i = 1, ..., 1000\}$. We randomly sample a subset of 8,424 vitrimers from the whole dataset and calculate their T_g by MD simulations and GP calibration. A subset of 7,424 vitrimers is denoted as the labeled training set $\mathcal{D}_\text{prop} = \{(\mathcal{G}^{\text{a}(i)}, \mathcal{G}^{\text{e}(i)}, T_\text{g}^{(i)}) : i = 1, ..., 7424\}$ and the rest 1,000 vitrimers constitute the test set $\mathcal{D}_\text{test}$. $\mathcal{D}$ and $\mathcal{D}_\text{prop}$ are used to train the VAE on a two-step basis (see Equation 7 and Equation 9 in the manuscript) and $\mathcal{D}_\text{test}$ allows for evaluation of the reconstruction and property prediction capabilities on unseen data. The network dimensions and hyperparameters of the VAE framework are presented in Table S1,S2, respectively. Note that we do not differentiate between the acid and epoxide encoders (decoders) because the network architectures are identical.

S3.3 VAE performance

The metrics of the VAE model before and after joint training with the small dataset $\mathcal{D}_\text{prop}$ are presented in Table S3. The VAE model is trained three times with different training/test dataset splits and random initializations. For each metric, the mean and standard deviations are reported. The uncertainty in all metrics is low, confirming the robustness of our model under different conditions. The improved metrics after joint training show that the model is not biased to $\mathcal{D}_\text{prop}$ and is able to accommodate and generate a wide range of vitrimers. Examples of ten vitrimers from the test set and their reconstructions are shown in Figure S6. Two out of ten vitrimers are not successfully reconstructed due to mismatch of one of the components. Figure S7 presents examples of 20 vitrimers sampled from the latent space based on standard Gaussian distribution. The three invalid vitrimers have chemically valid acids or epoxides which are not bifunctional. The predicted T_g by the property predictor is compared with calibrated T_g for 1,000 vitrimers in the test set, as shown in Figure S8. A low MAE of 13.53 K indicates high accuracy of the trained property predictor. The distributions of latent vectors encoded from the labeled training set $\mathcal{D}_\text{prop}$ and test set $\mathcal{D}_\text{test}$ after joint training are shown in Figure S9a,b. Compare with the distributions before joint training (Figure S9c,d), the gradient in T_g is much more recognizable, proving the effect of latent space organization by joint training the VAE with property predictor.

S3.4 Exploration of latent space

Starting with the latent vector of a know vitrimer $\boldsymbol{z_0}$ as origin, we can find novel vitrimers by adding noise, i.e.,

$$\boldsymbol{z} = \boldsymbol{z}_0 + \beta \cdot \boldsymbol{\epsilon}, \tag{12}$$

where β determines the magnitude of the noise and $\boldsymbol{\epsilon} \sim \mathcal{N}(\mathbf{0}, \boldsymbol{I})$. The partial overlapping method enables us to explore the neighborhood along three axes: acid-specific (first d_a dimensions of $\boldsymbol{\epsilon}$ are non-zero), epoxide-specific (last d_e dimensions of $\boldsymbol{\epsilon}$ are non-zero) and both (all dimensions of $\boldsymbol{\epsilon}$ are non-zero).

We define a spherical interpolation (SLERP) path between two latent representations $\boldsymbol{z}_1$ and $\boldsymbol{z}_2$:

$$\mathrm{SLERP}(\boldsymbol{z}_1, \boldsymbol{z}_2; \alpha) = \frac{\sin((1-\alpha)\theta)}{\sin\theta}\boldsymbol{z}_1 + \frac{\sin(\alpha\theta)}{\sin\theta}\boldsymbol{z}_2, \tag{13}$$

where $\alpha \in [0, 1]$ is the interpolation parameter and θ is the angle between $\boldsymbol{z}_1$ and $\boldsymbol{z}_2$. This is different from the linear interpolation (LERP) that has been used in previous works [15, 16]:

$$\mathrm{LERP}(\boldsymbol{z}_1, \boldsymbol{z}_2; \alpha) = (1-\alpha)\boldsymbol{z}_1 + \alpha\boldsymbol{z}_2. \tag{14}$$

We choose SLERP over LERP since LERP operates on the presumption of a linear connection between points which disregards the inherent structure of the multi-dimensional Gaussian distribution. Moreover, LERP calculates the Euclidean distance between two points which may not be consistent with the similarity between vitrimers in the latent space. Therefore, points along the linear interpolation path might leap across regions within the latent space that share similar molecular structures which results in irregular or unnatural interpolations. On the other hand, SLERP acknowledges the hyperspherical structure of the latent space and pursues the shortest arc on the surface of a d-dimensional hypersphere, thereby minimizing unrealistic transitions along the path. Figure S10 shows the decoded vitrimers along SLERP and LERP paths, their locations in the latent space and T_g. Previous studies [17, 18] have addressed the effectiveness of SLERP, which is more suitable for traversing between two locations in the latent space created by VAEs.

S3.5 Bayesian optimization

We use the batch Bayesian optimization algorithm developed by Kusner et al. [19]. The workflow of Bayesian optimization is illustrated in Figure S11. We start with 1,000 latent vectors $\boldsymbol{z}$ randomly sampled from standard Gaussian distribution and decode them into valid vitrimers. These vitrimers are then encoded into reconstructed latent vectors and their T_g is predicted by the property predictor. The predicted T_g is converted into the optimization objective, which is either $-T_\mathrm{g}$ (if the task is to find vitrimers with the maximum T_g) or the squared error between T_g and the target T_g (if the task is to find vitrimers with a target T_g). In each iteration, a sparse Gaussian process with 100 inducing points is trained as a surrogate model and 50 vitrimers are proposed by the expected improvement acquisition function. The valid vitrimers and their predicted T_g are added to the training set for next iteration. Ten independent runs each with 50 iterations are performed and all valid proposed vitrimers are collected. For each of the three design targets, we select 100 candidates with predicted T_g closest to the target that can be parameterized by PCFF, carry out MD simulations and calibrate MD-calculated T_g^MD. The distributions of T_g of the designed vitrimers and the dataset are presented in Figure S12. Ten best candidates with T_g closet to each target are presented in Figure S13. Our framework succeeds in extrapolating beyond the training regime and achieves accurate inverse design within an error of 2 K.

To examine the stability of vitrimers presented in Figure S13, we minimize them under reactive force field (ReaxFF). ReaxFF is a molecular dynamics simulation technique that captures the instantaneous interactions between atoms using the bond order concept [20]. This strategy allows for a smooth transition between the non-bonded and bonded configurations and helps with the investigation of chemical reactions where bond formation and dissociation are involved. The proposed molecules remain intact during minimization

using the CHON2017_weak_bb force field [21] and the minimized structures are presented in Figure S14. The chemical space covered by the calibration dataset and proposed vitrimers is visualized by PCA of fingerprints in Figure S15. We further calculate relevant molecular descriptors of these vitrimers, as shown in Figure S16. Density is extracted from MD simulations at 300 K. All other descriptors are calculated by the Mordred package [22]. Each value is calculated from the repeating units (Figure S1a with $n = 1$) and averaged over ten proposed vitrimers.

We compare the T_g of the proposed vitrimers with commonly used polymers in Figure S17. The vitrimers from inverse design cover a wide range of T_g from around 250 K to 550 K. With further tuning of the design target, our framework has the potential to discover vitrimers with any T_g within the range and greatly enhances the applicability of vitrimers at various temperatures.

S4 Computational efficiency

The computational runtime, used software and hardware for different tasks are listed in Table S4 to demonstrate the efficiency of the proposed method in this work. The trained property predictor serves as a shortcut for costly MD simulations to estimate T_g of vitrimers, which also allows for efficient discovery of novel vitrimers with target T_g by Bayesian optimization.

S5 Experimental synthesis and characterization

The synthesis of the proposed vitrimer (Figure 6 in the manuscript) is performed by a two-step reaction. The first step involves mixing of succinic anhydride (1 mol) with glycerol (0.5 mol) at 130 °C for 1 hour to obtain a clear solution mixture of polymer with bifunctional carboxylic acid groups. In the second step, equivalent stoichiometric amount of DGEBA is added to the mixture in presence of catalyst triazabicyclodecene (TBD), which is further mixed at the same condition for 30 minutes to obtain a homogeneous slurry. The slurry is then transferred into a preheated Teflon taped mold at 145 °C and covered with top cover. The mold is placed into the heat press to continue the crosslinking at 145 °C for 6 hours followed by post curing for 2 hours at 180 °C.

The crosslinked vitrimer specimen is verified using Fourier-transform infrared (FTIR) spectroscopy, as shown in Figure S18. Differential scanning calorimetry (DSC) is carried out by TA Instruments Q2000. Thermomechanical analysis (TMA) is performed at 0.1 N applied force with a heating rate of 5 °C per minute using Perkin Elmer TMA7. Tensile tests are carried out at 2 mm/min deformation rate with a sample dimension of 50 mm $\times$ 5 mm $\times$ 1.5 mm. Three replicate tests are done and the results are presented in Figure S19 and Table S5. To demonstrate the recyclability of the synthesized vitrimer (Figure S20), a sample is cut into pieces and heat pressed under 180 °C and 1 MPa for an hour. The recovered sample confirms the reprocessability and recyclability of the synthesized vitrimer.

Another round of Bayesian optimization is performed to design vitrimers with a higher target T_g of 373 K. The epoxide is fixed as DGEBA and the model proposes a commercially available acid 1,4-cyclohexanedicarboxylic acid (CHDA). The vitrimer composed of CHDA and DGEBA has been synthesized in a previous work [23] and the measured T_g is 358 ± 2.3 K, which aligns well with the design target and validates the efficacy of our framework in designing vitrimers with higher target T_g (Figure S21).

Hyperparameters	Encoder $\mathcal{Q}_\phi$	Decoder $\mathcal{P}_\theta$	Property predictor $\mathcal{F}_\omega$
Input dimensions	none	$d_a = d_e = 112$	$d = 128$
Embedding dimensions	250	250	none
Hidden dimensions	250	250	64
Output dimensions	$d_a = d_e = 112$	none	1

Table S1: Hyperparameters of the network architecture.

Hyperparameters	Step one	Step two
λ_{KL}	0.005	0.005
Batch size	32	32
Optimizer	Adam [24]	Adam [24]
Learning rate	constant 0.001	$0.001 \times 0.9^{i-1}$ at epoch i
Number of epochs	10	50

Table S2: Hyperparameters of the training protocols.

Metrics	Before joint training	After joint training
Reconstruction accuracy	87.3% ± 4.1%	89.1% ± 2.8%
ample validity	80.9% ± 2.5%	82.9% ± 2.0%
Sample novelty	100.0% ± 0.0%	100.0% ± 0.0%
Sample uniqueness	100.0% ± 0.0%	100.0% ± 0.0%

Table S3: Metrics of the VAE before and after joint training.

Tasks	Software	Hardware	Runtime (h)
T_g calculation of one vitrimer by MD	LAMMPS	CPU	310
Training of the GP model and calibrating T_g of 8,424 vitrimers	Python	CPU	0.006
Training of the VAE with $\mathcal{D}$	PyTorch	GPU	130
Joint training of the VAE and property predictor $\mathcal{F}_\omega$ with $\mathcal{D}_{prop}$	PyTorch	GPU	5
T_g prediction of 1,000 vitrimers by trained $\mathcal{F}_\omega$	PyTorch	GPU	0.01
Proposing ∼ 1,300 candidate vitrimers by Bayesian optimization	PyTorch	GPU	1

Table S4: The computational runtime, used software and hardware for different tasks in this work. The presented runtime is roughly estimated with one core of a 2.1 GHz Intel Xeon Gold 6230 CPU and a NVIDIA GeForce RTX 2080 Ti GPU.

	Tensile strength (MPa)	Elastic modulus (MPa)
Test 1	12.76	774.26
Test 2	10.14	658.39
Test 3	10.17	643.17
Average	11.02 ± 1.50	691.94 ± 71.70

Table S5: Mechanical properties of the synthesized vitrimer.

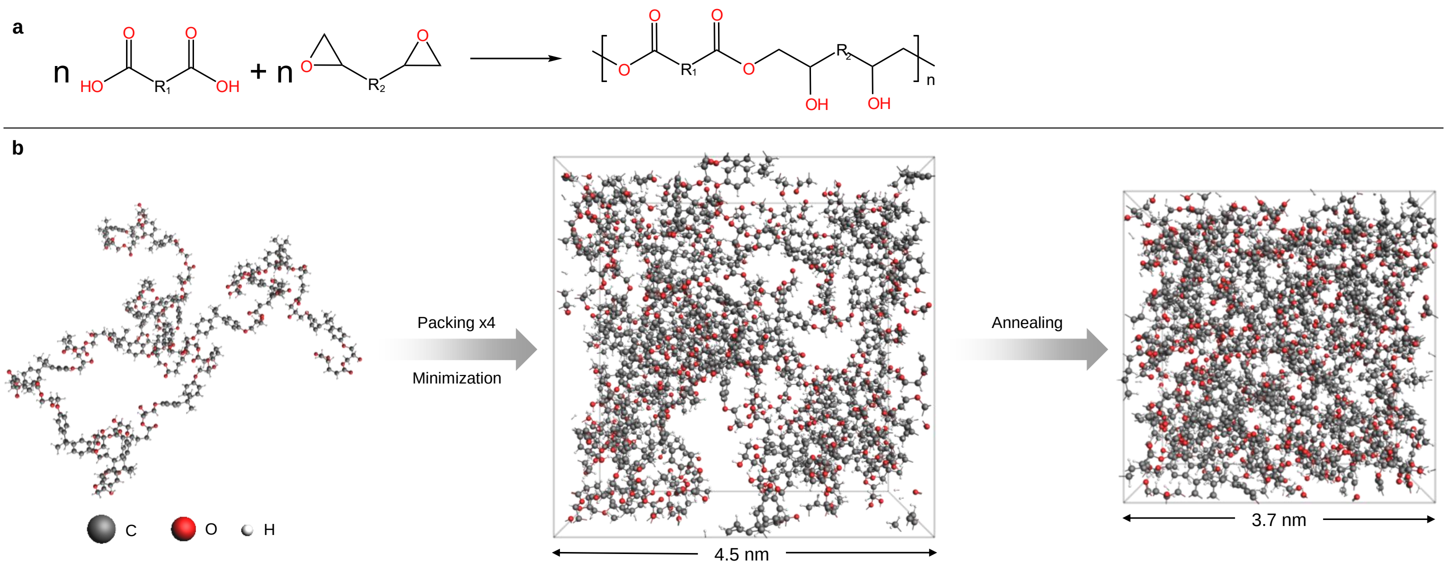

Figure S1: Molecular dynamics simulations to calculate T_g of vitrimers. a) A virtual vitrimer chain is made by connecting carboxylic acids and opened epoxides in an alternating manner. b) Four chains of $\sim$ 1,000 atoms are placed in a simulation box. The system is annealed to remove local heterogeneities before production.

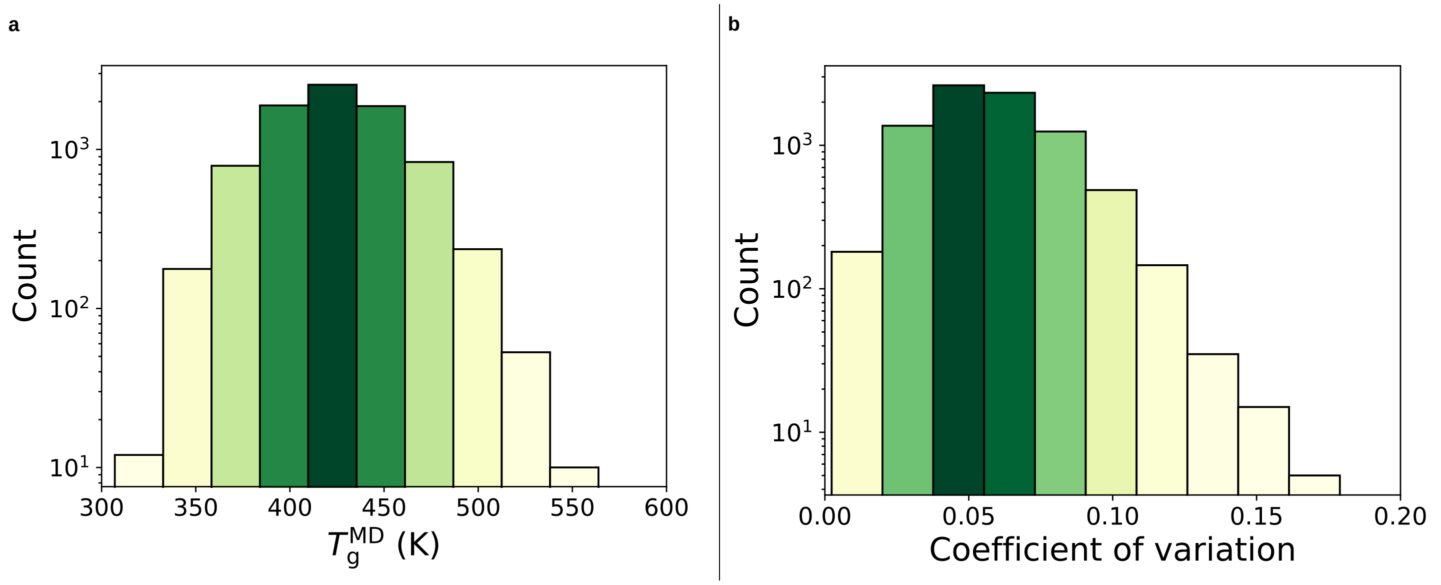

Figure S2: Distributions of a) mean T_g and b) coefficient of variation in T_g from five replicate MD simulations of 8,424 vitrimers.

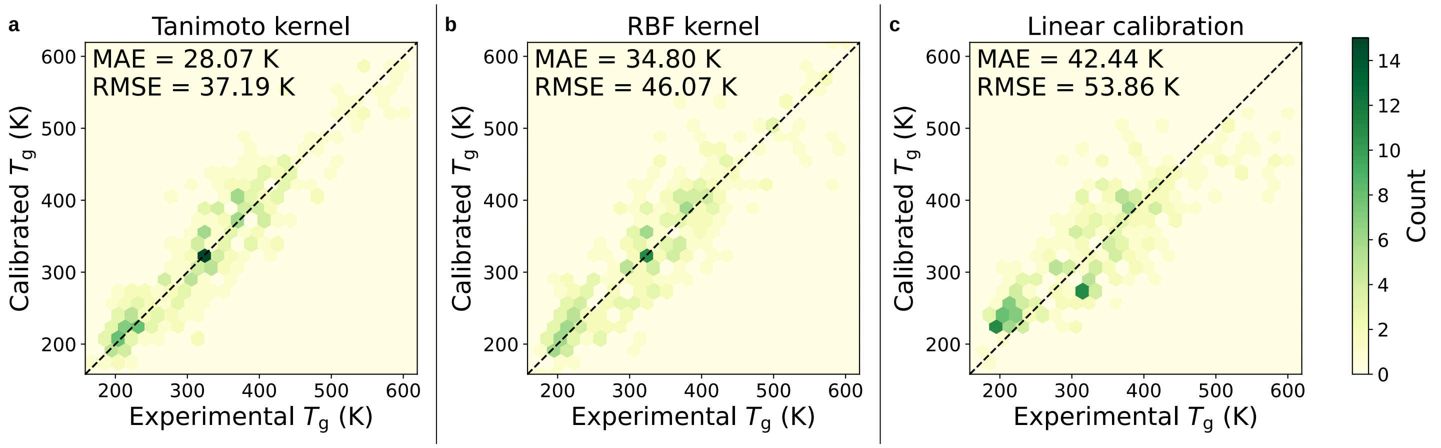

Figure S3: Calibrated T_g vs. experimental T_g by a) GP with Tanimoto kernel, b) GP with RBF kernel and c) linear regression.

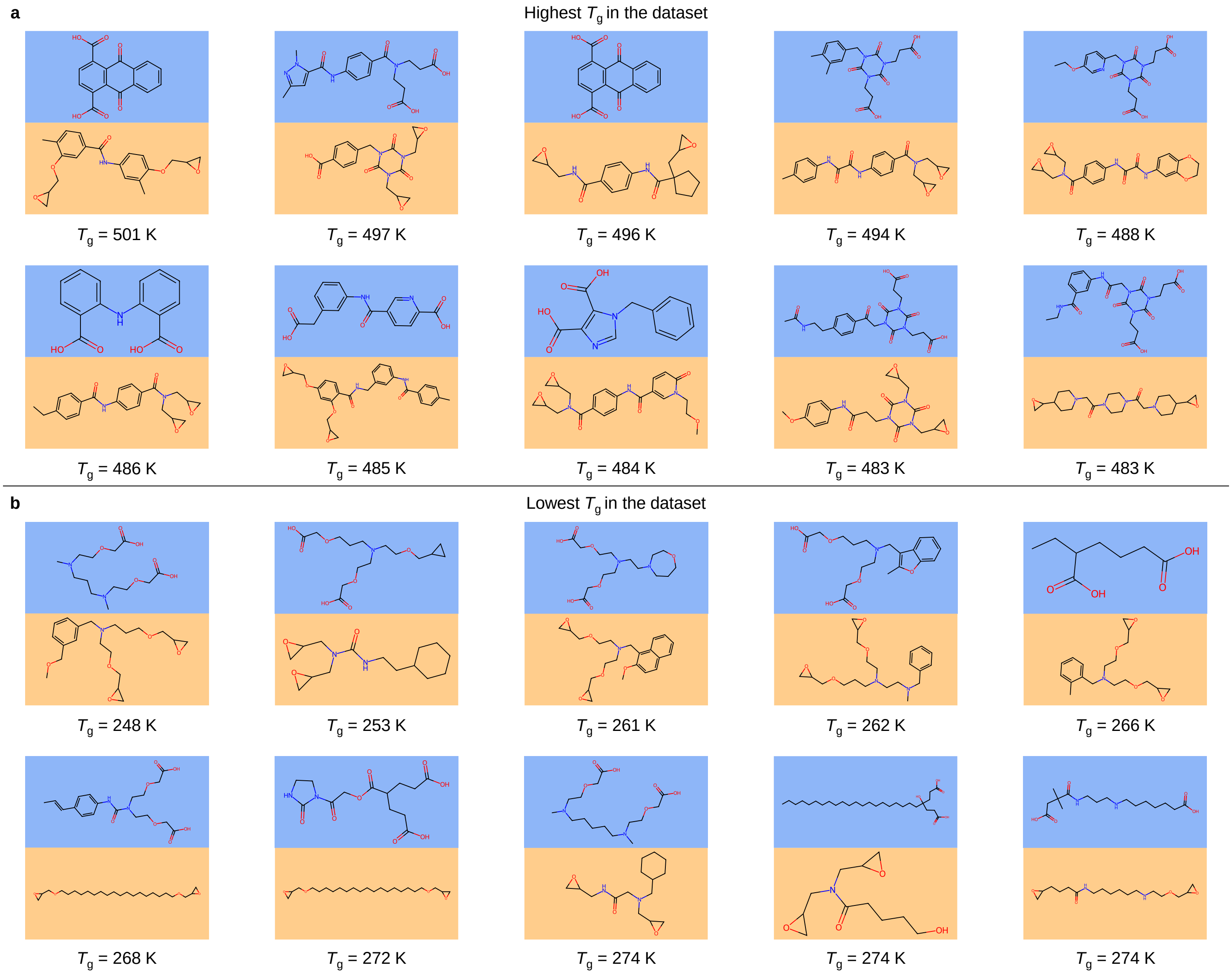

Figure S4: Ten example vitrimers with highest and lowest T_g in the dataset.

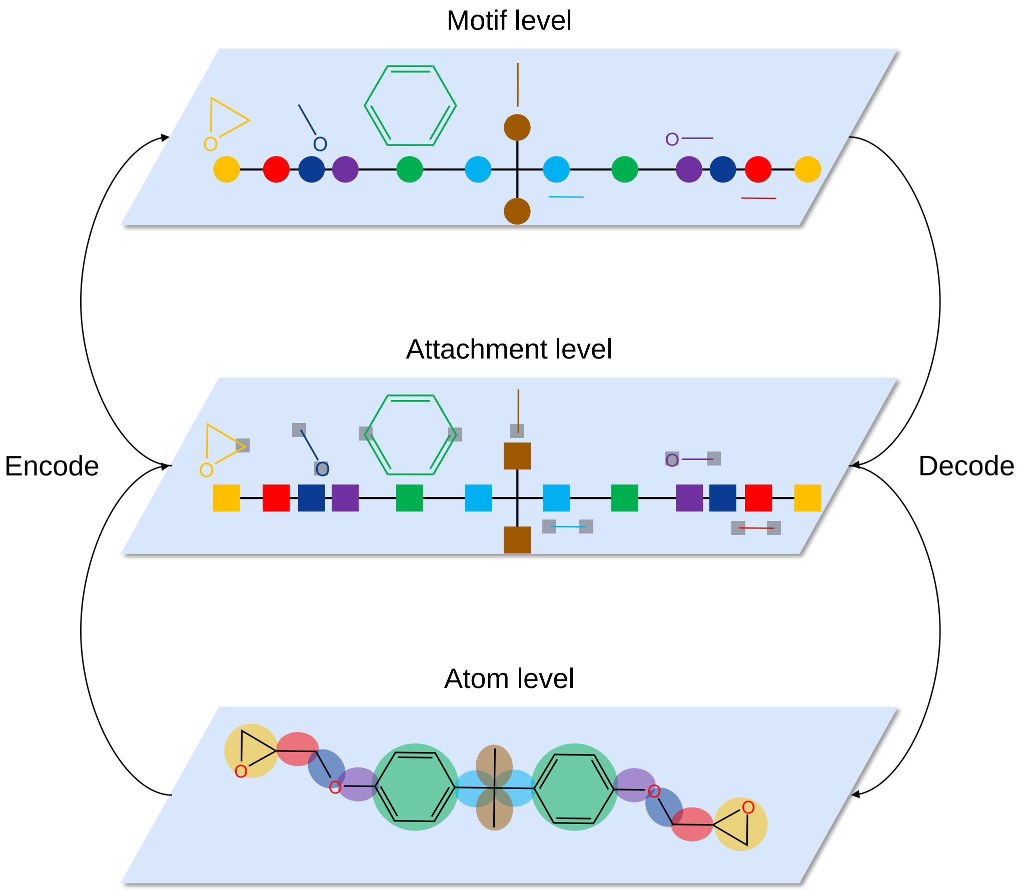

Figure S5: Schematic illustration of the three-level hierarchical graph representation. The shared atoms between motifs are highlighted in grey blocks on the attachment level.

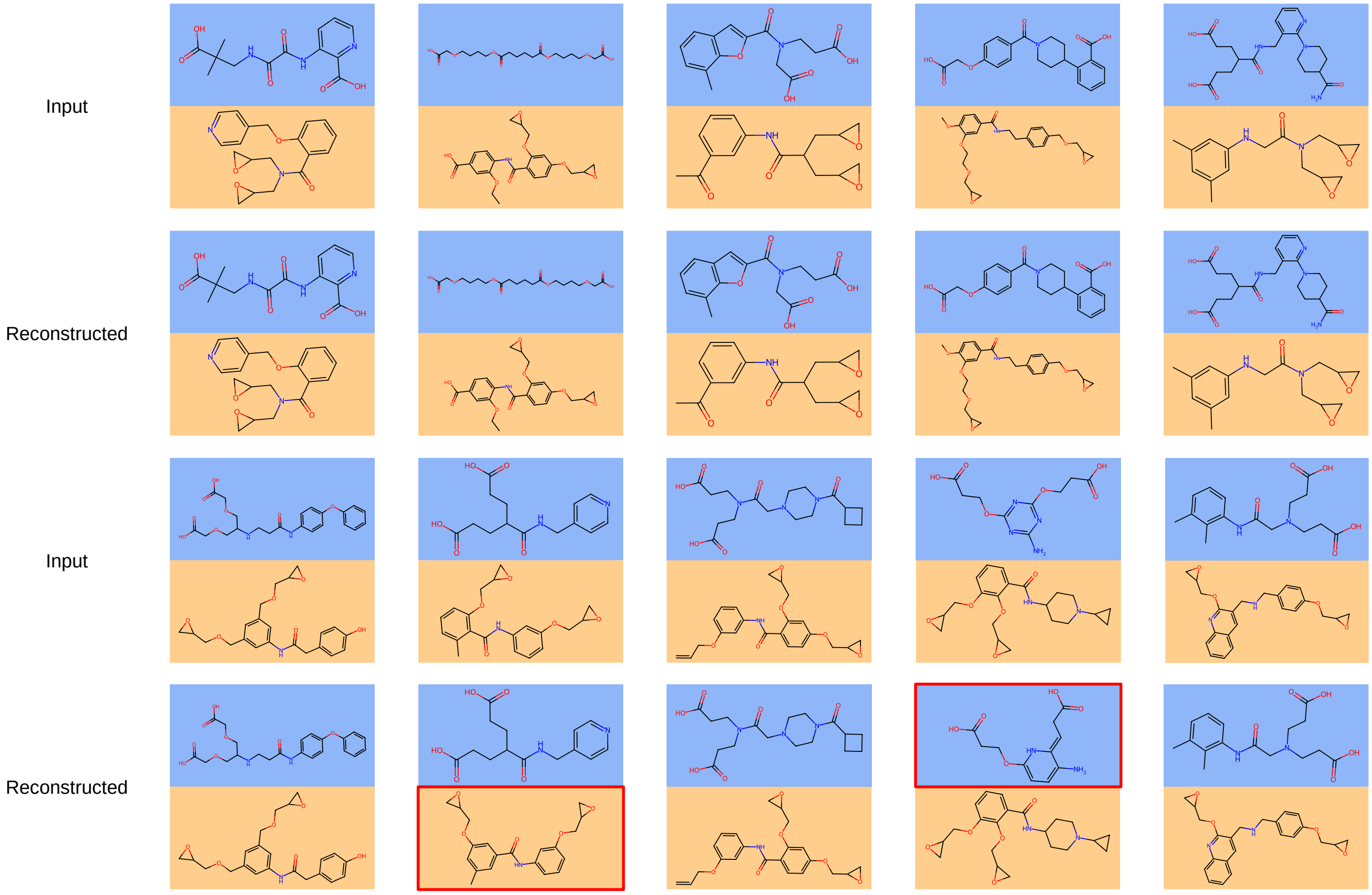

Figure S6: Examples of input and reconstructed vitrimers from the test set. Unsuccessful reconstructions are highlighted in red boxes.

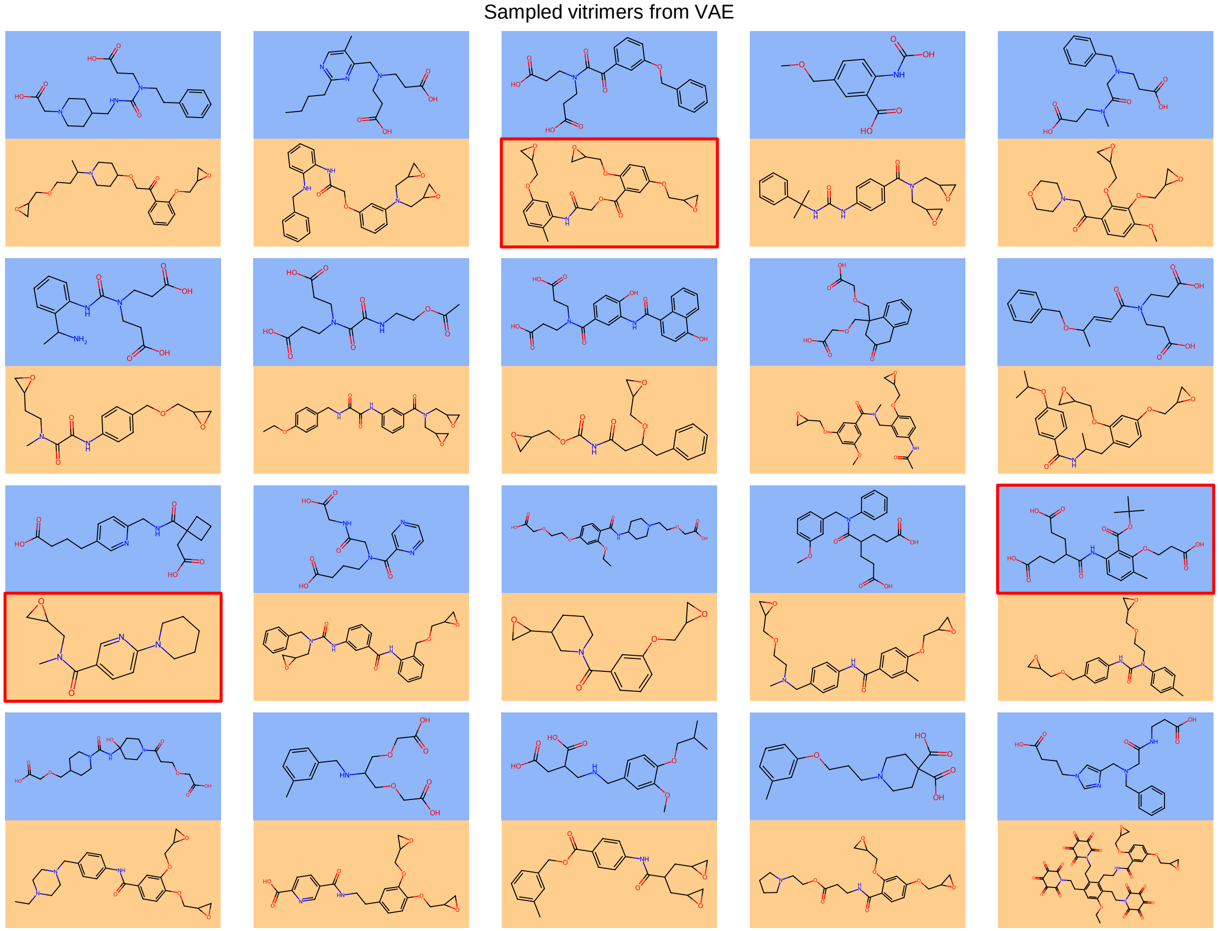

Figure S7: Examples of vitrimers sampled from the latent space according to standard Gaussian distribution. Invalid vitrimer components are highlighted in red boxes.

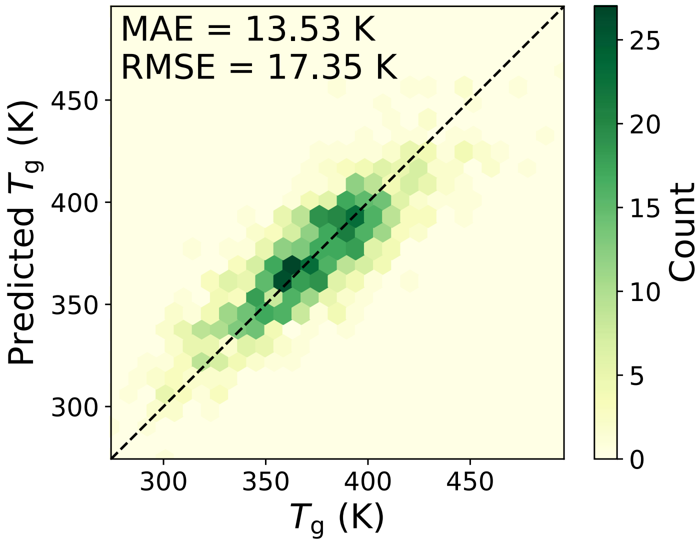

Figure S8: Predicted T_{g} by the property predictor vs. true T_{g}.

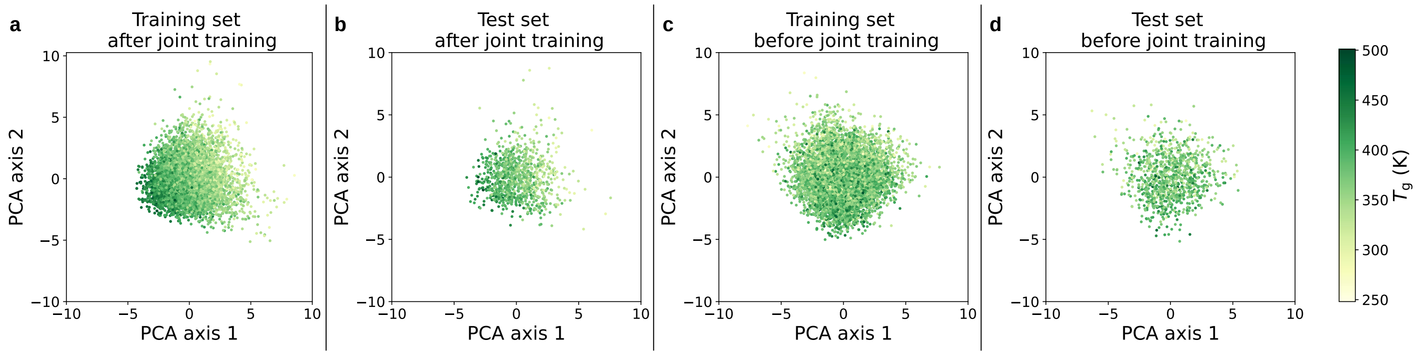

Figure S9: Distributions of latent vectors of a) training set after joint training, b) test set after joint training, c) training set before joint training and d) test set before joint training. Latent vectors of higher dimensions are projected into two principal axes for visualization using principal component analysis (PCA).

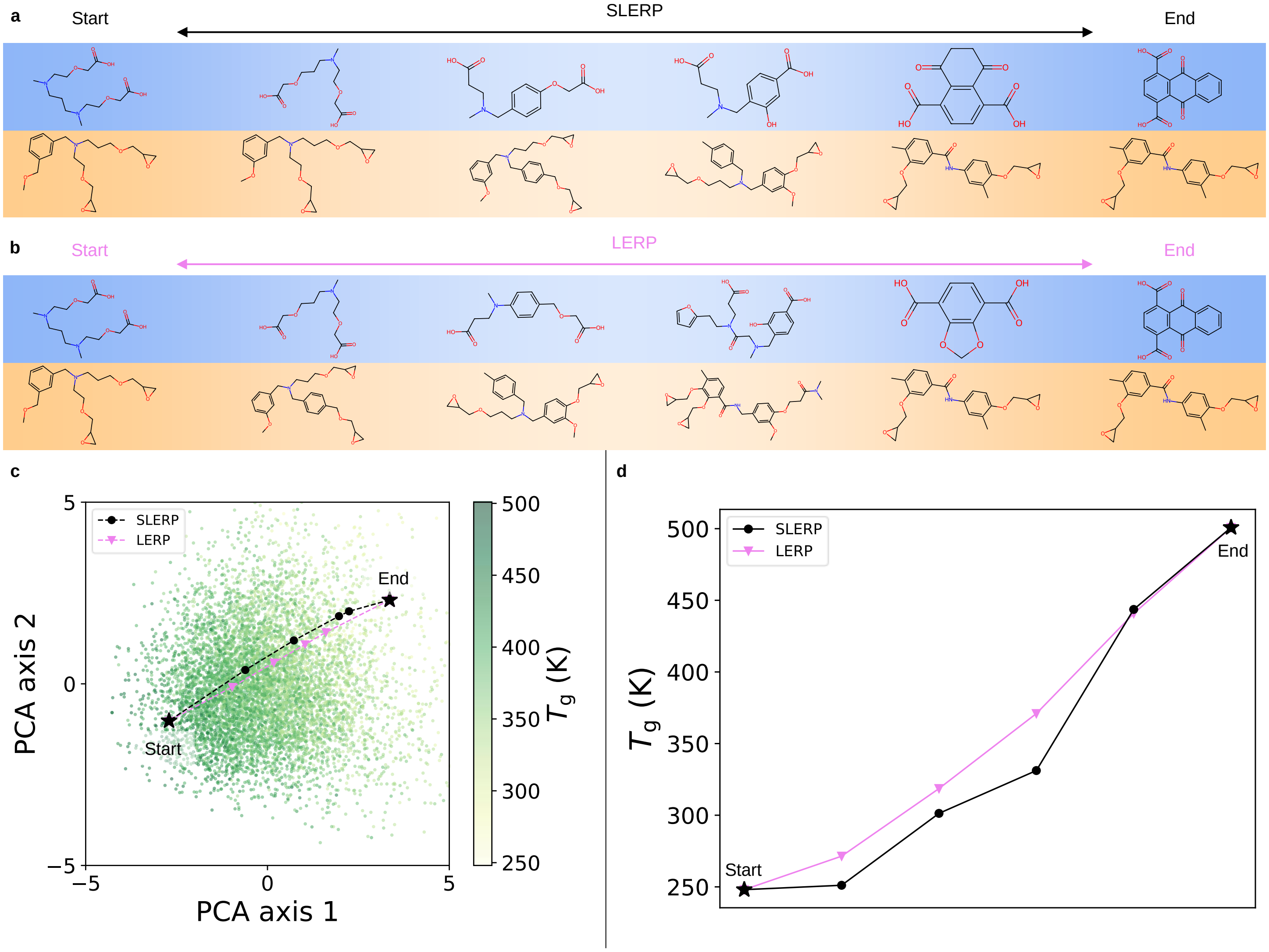

Figure S10: Comparison between spherical interpolation (SLERP) and linear interpolation (LERP). Vitrimers are discovered by a) SLERP and b) LERP in the latent space. c) Interpolation paths in latent space visualized by PCA. d) T_g of discovered vitrimers. All presented T_g values of are validated by MD simulations and GP calibration.

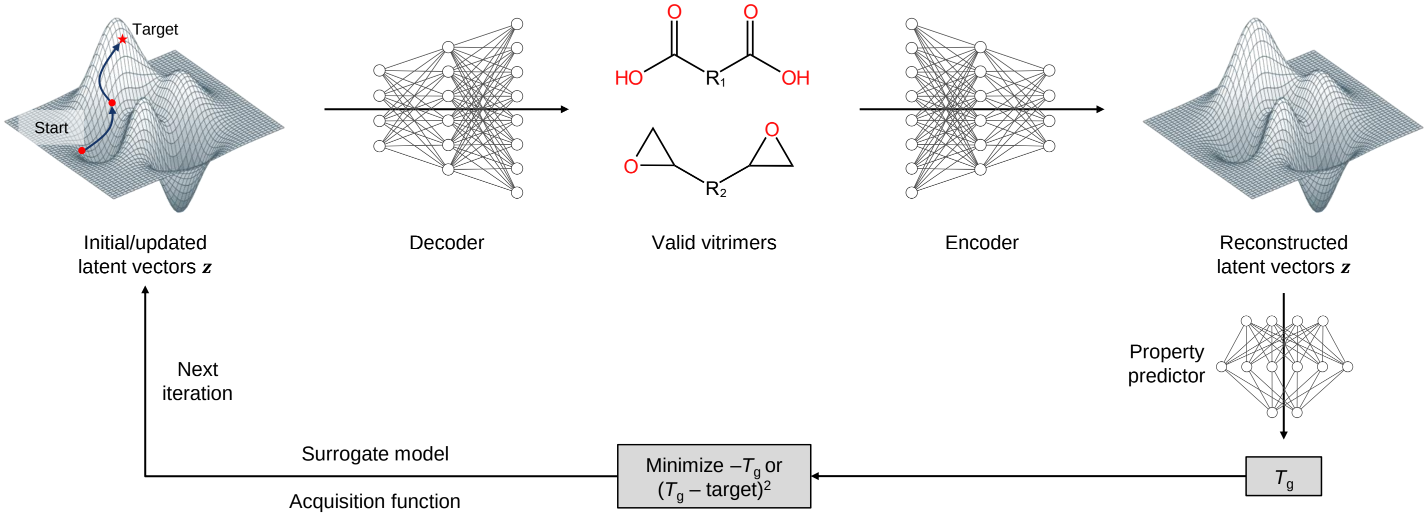

Figure S11: Schematic workflow of Bayesian optimization.

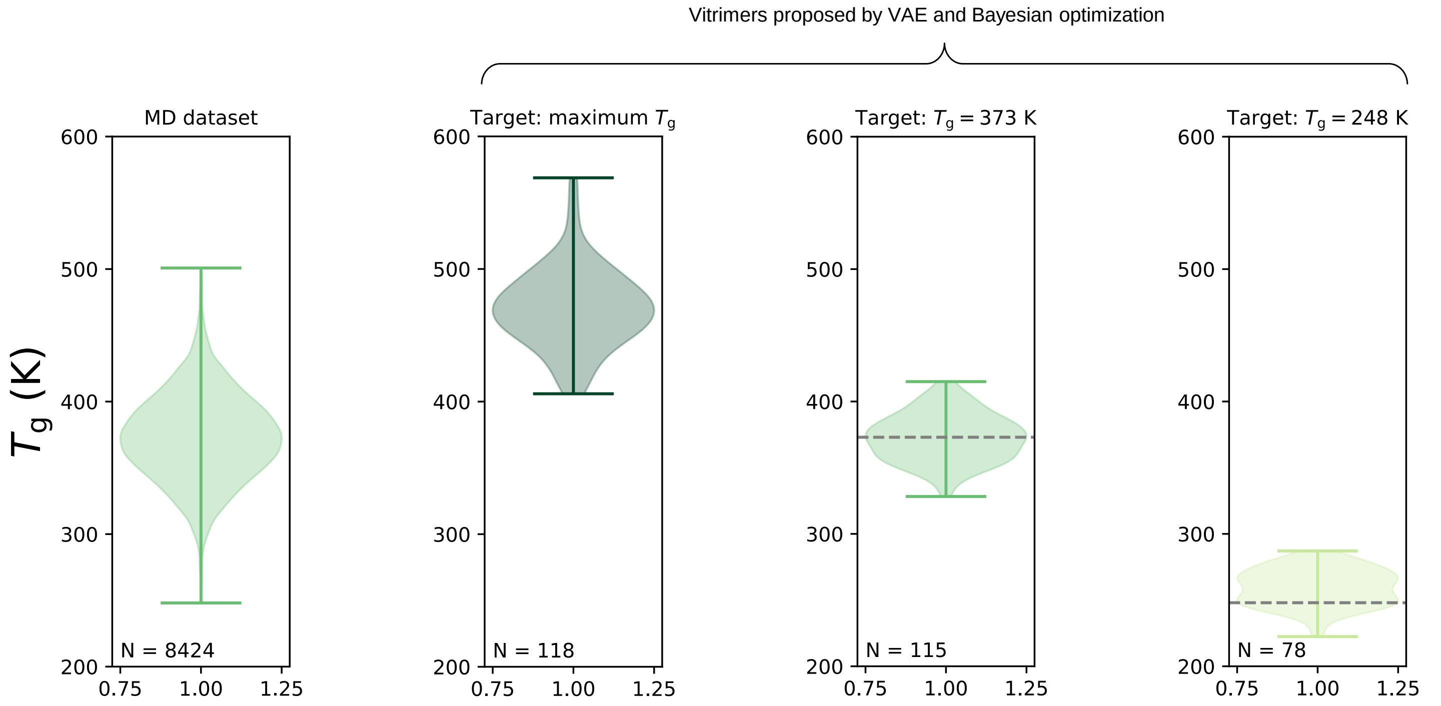

Figure S12: Distributions of T_{g} of the dataset and the vitrimers proposed by the VAE and Bayesian optimization. All presented T_{g} values of are validated by MD simulations and GP calibration.

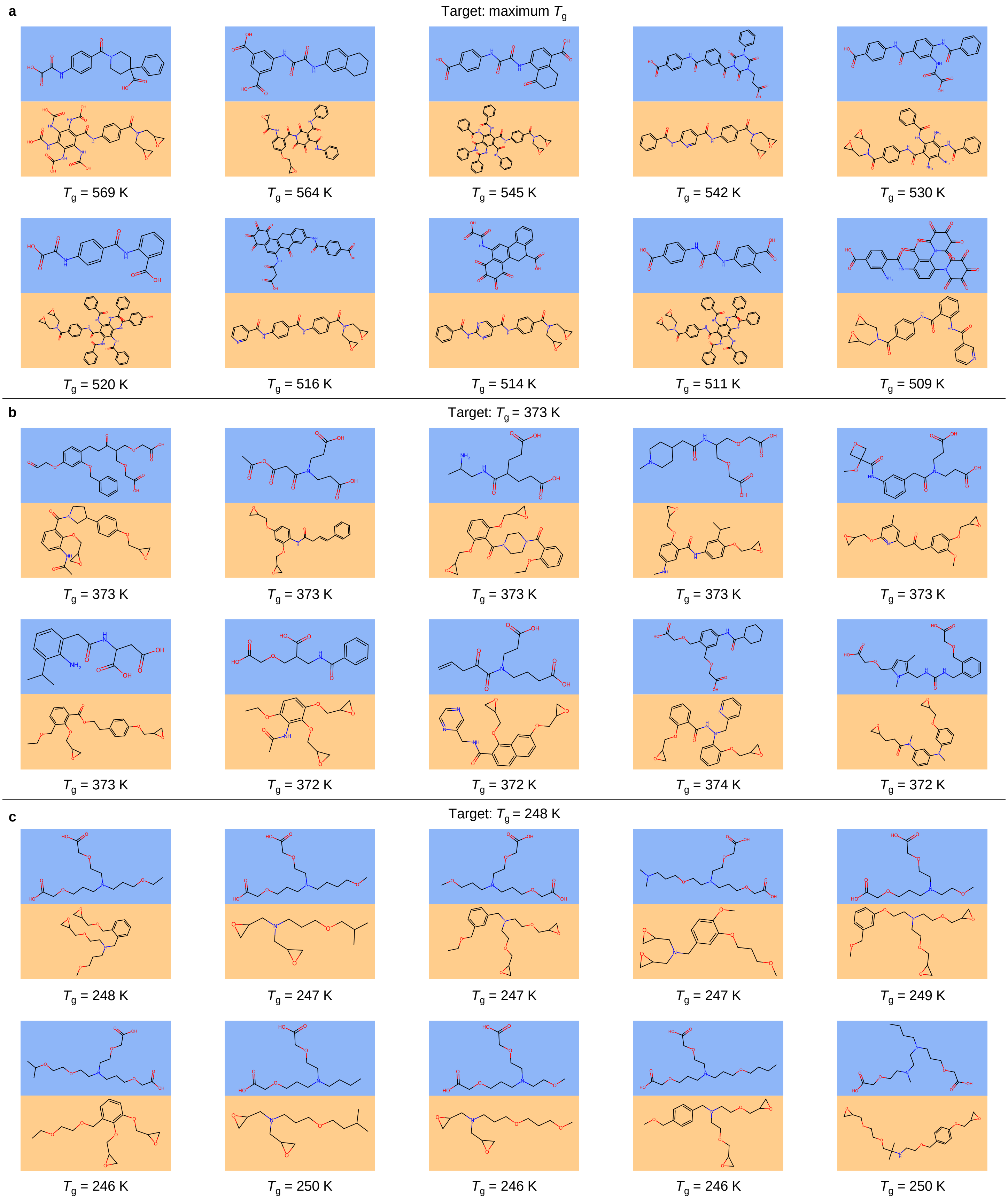

Figure S13: Examples of novel vitrimers designed with different target T_g from Bayesian optimization. a) Maximum T_g. b) Target $T_g = 373$ K. c) Target $T_g = 248$ K. All presented T_g values of proposed vitrimers are validated by MD simulations and GP calibration.

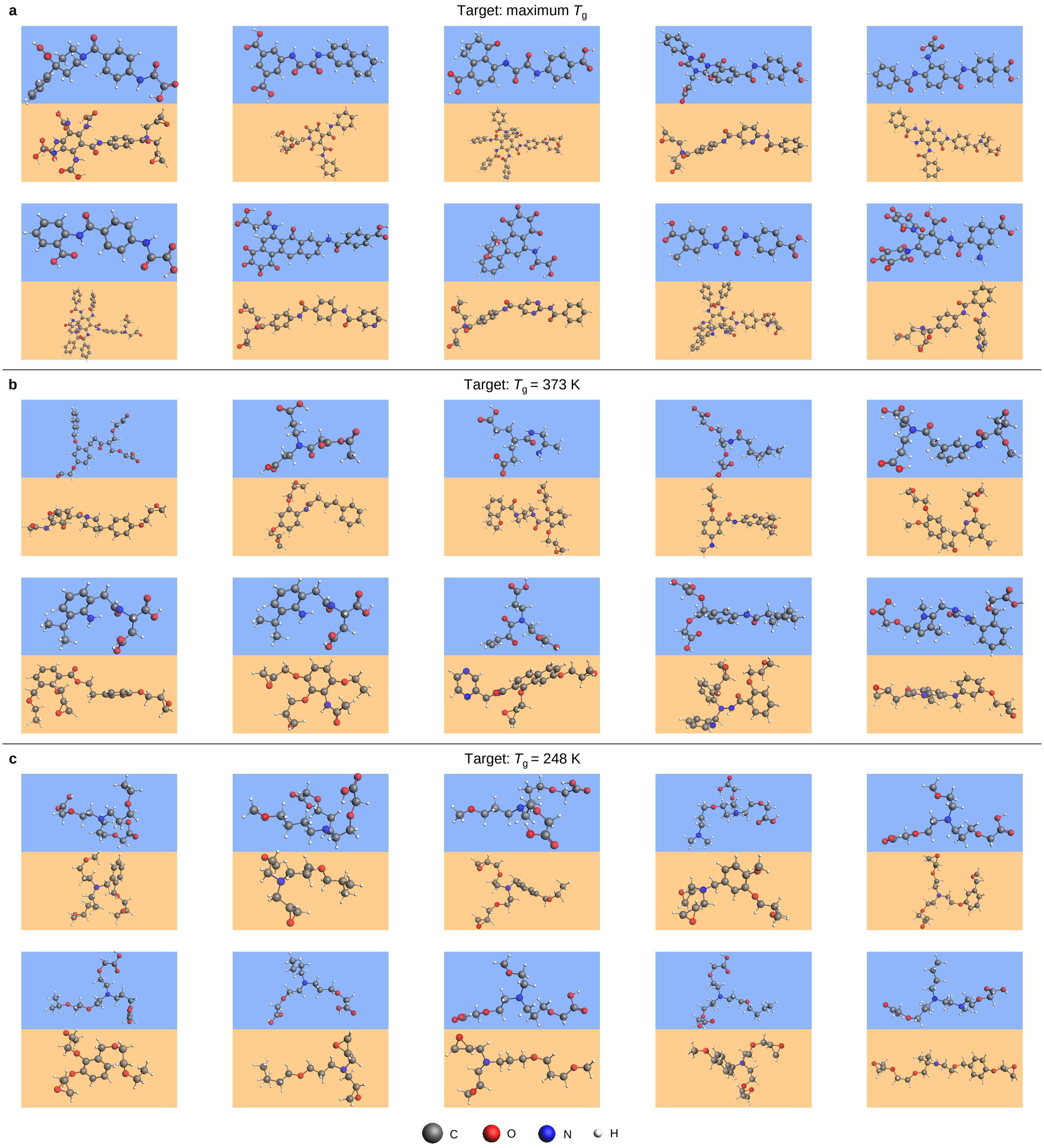

Figure S14: Minimized structures of vitrimer components presented in Figure S13. The molecules are minimized by ReaxFF using the CHON2017_weak_bb force field.

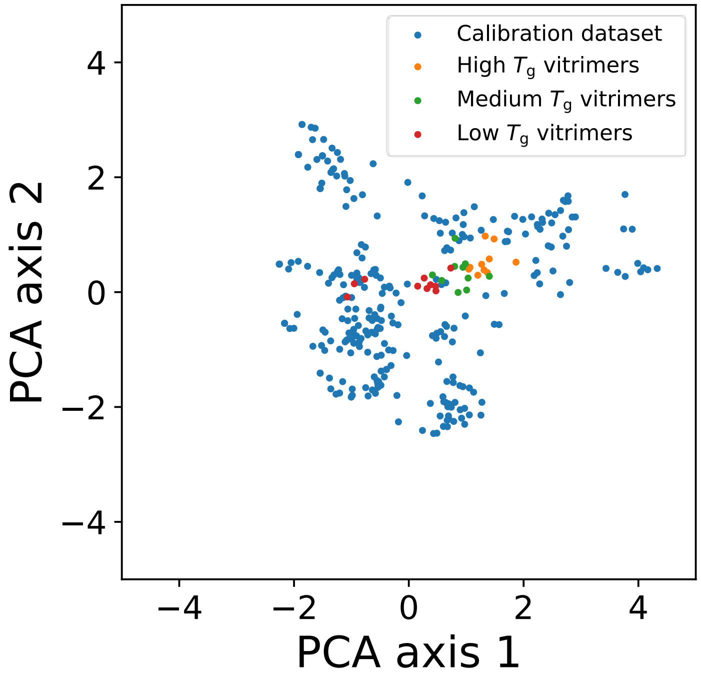

Figure S15: Reduced molecular fingerprints of 295 polymers in the calibration dataset and 30 proposed vitrimers for three T_g targets in two-dimensional space by PCA.

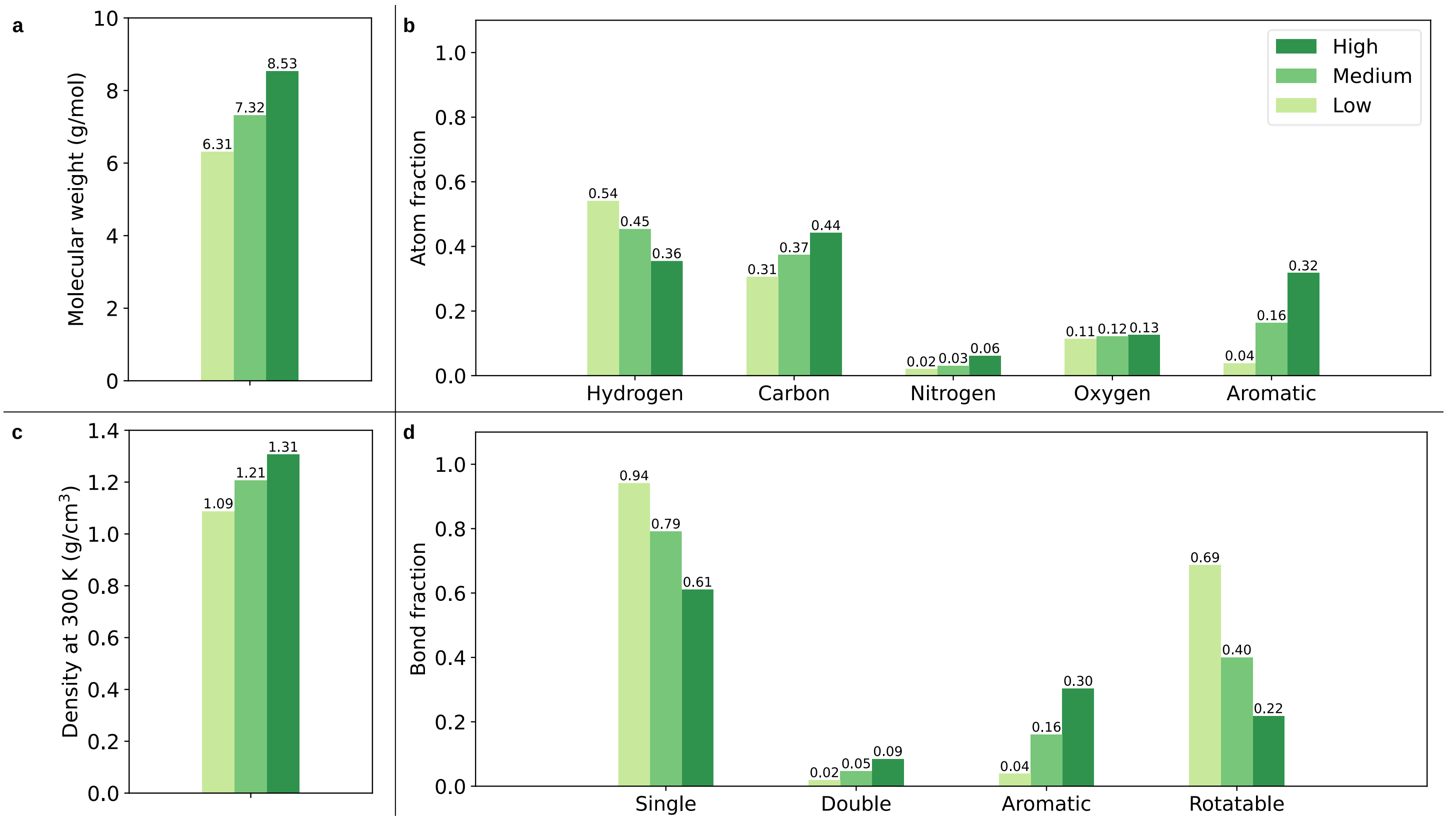

Figure S16: Relevant molecular descriptors of the vitrimers presented in Figure S13. a) Molecular weight. b) Atom fractions. c) Density at 300 K. d) Bond fractions. All values are averages of ten vitrimers per target.

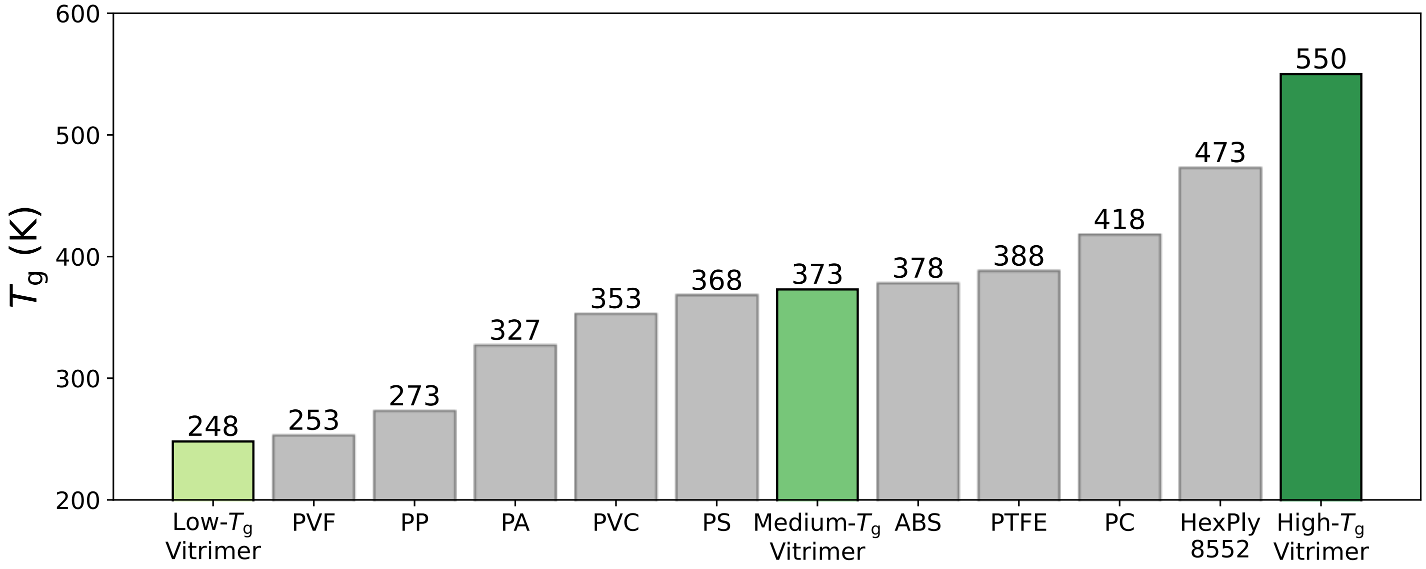

Figure S17: T_g of commonly used polymers and our proposed vitrimers by inverse design.

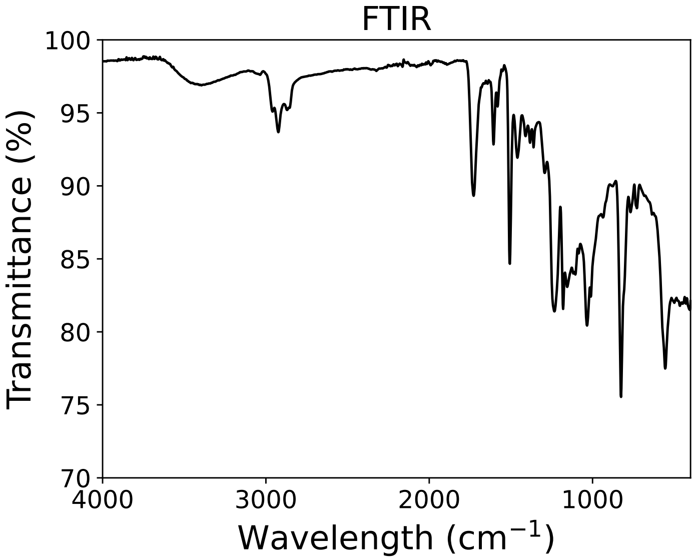

Figure S18: Fourier-transform infrared (FTIR) spectroscopy of the synthesized vitrimer.

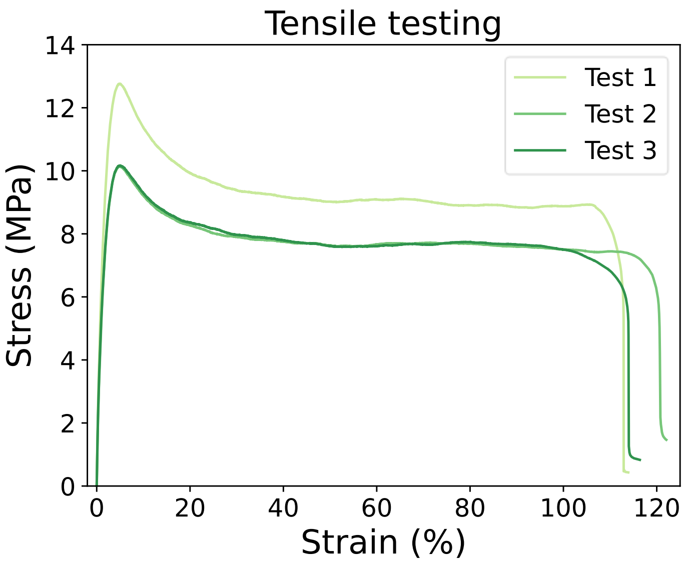

Figure S19: Tensile testing of the synthesized vitrimer.

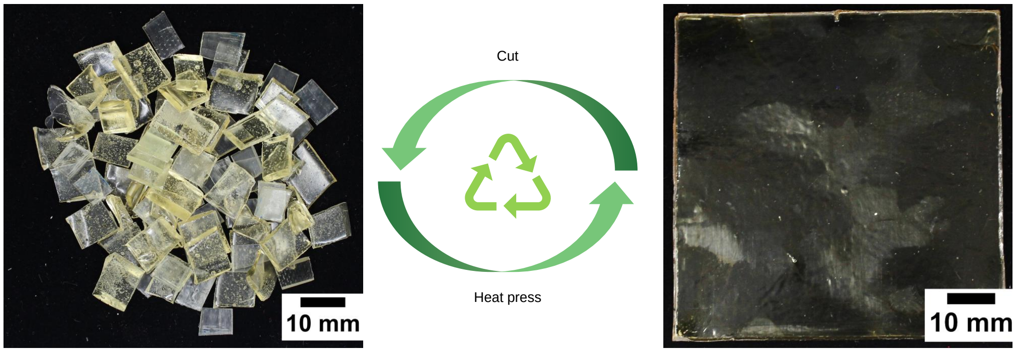

Figure S20: Recycling the cut vitrimer sample by heat press.

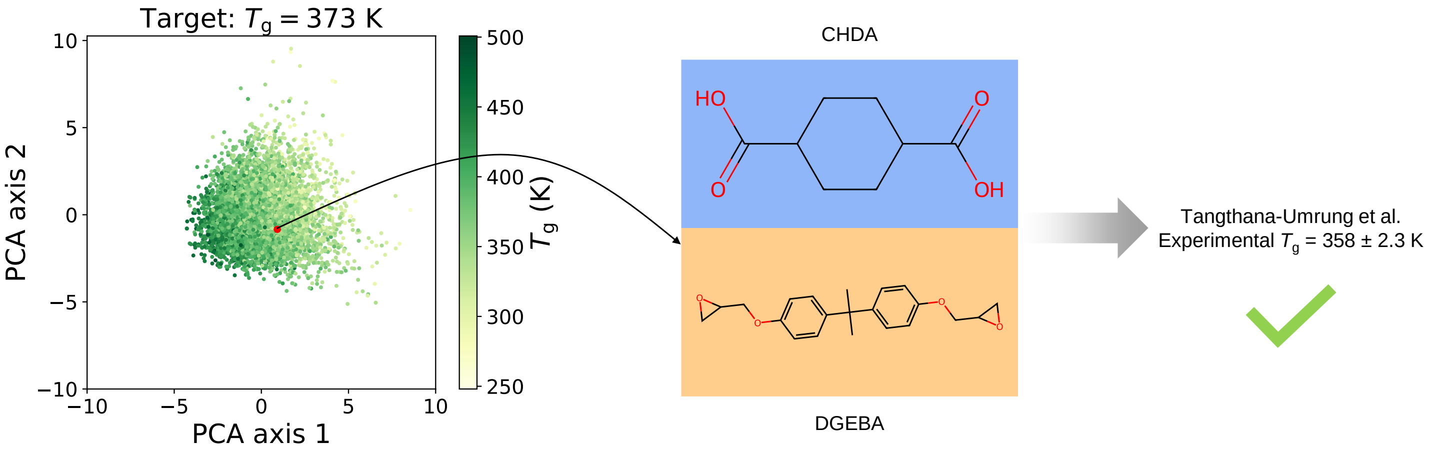

Figure S21: Inverse design of vitrimer with a higher target $T_{\mathrm{g}} = 373$ K. The model proposes a vitrimer composing of 1,4-cyclohexanedicarboxylic acid (CHDA) and bisphenol A diglycidyl ether (DGEBA). This vitrimer has been synthesized in a previous work [23] and the measured T_{g} is 358 ± 2.3 K, validating the efficacy of our framework when targeting a higher T_{g}.

References

[1] A. P. Thompson, H. M. Aktulga, R. Berger, D. S. Bolintineanu, W. M. Brown, P. S. Crozier, P. J. in't Veld, A. Kohlmeyer, S. G. Moore, T. D. Nguyen, et al., *Computer Physics Communications* **2022**, *271* 108171.

[2] H. Sun, S. J. Mumby, J. R. Maple, A. T. Hagler, *Journal of the American Chemical society* **1994**, *116*, 7 2978.

[3] Y. Sun, H. Yang, K. Yu, Y. Guo, J. Qu, *Polymer* **2019**, *180* 121702.

[4] C. Park, G. Kim, J. Jung, B. Krishnakumar, S. Rana, G. J. Yun, *Polymer* **2020**, *206* 122862.

[5] P. J. in't Veld, G. C. Rutledge, *Macromolecules* **2003**, *36*, 19 7358.

[6] L. Alzate-Vargas, M. E. Fortunato, B. Haley, C. Li, C. M. Colina, A. Strachan, *Modelling and Simulation in Materials Science and Engineering* **2018**, *26*, 6 065007.

[7] M. A. F. Afzal, A. R. Browning, A. Goldberg, M. D. Halls, J. L. Gavartin, T. Morisato, T. F. Hughes, D. J. Giesen, J. E. Goose, *ACS Applied Polymer Materials* **2020**, *3*, 2 620.

[8] D. Rogers, M. Hahn, *Journal of chemical information and modeling* **2010**, *50*, 5 742.

[9] L. Ralaivola, S. J. Swamidass, H. Saigo, P. Baldi, *Neural networks* **2005**, *18*, 8 1093.

[10] A. R. Thawani, R.-R. Griffiths, A. Jamasb, A. Bourached, P. Jones, W. McCorkindale, A. Aldrick, *ChemRxiv* **2020**.

[11] A. Jinich, B. Sanchez-Lengeling, H. Ren, R. Harman, A. Aspuru-Guzik, *ACS central science* **2019**, *5*, 7 1199.

[12] W. Jin, R. Barzilay, T. Jaakkola, In *International conference on machine learning*. PMLR, **2020** 4839–4848.

[13] A. Paszke, S. Gross, F. Massa, A. Lerer, J. Bradbury, G. Chanan, T. Killeen, Z. Lin, N. Gimelshein, L. Antiga, et al., *Advances in neural information processing systems* **2019**, *32*.

[14] G. Landrum, P. Tosco, B. Kelley, Ric, D. Cosgrove, sriniker, gedeck, R. Vianello, NadineSchneider, E. Kawashima, D. N, G. Jones, A. Dalke, B. Cole, M. Swain, S. Turk, AlexanderSavelyev, A. Vaucher, M. Wójcikowski, I. Take, D. Probst, K. Ujihara, V. F. Scalfani, guillaume godin, J. Lehtivarjo, A. Pahl, R. Walker, F. Berenger, jasondbiggs, strets123, Rdkit: Open-source cheminformatics., https://www.rdkit.org.

[15] R. Batra, H. Dai, T. D. Huan, L. Chen, C. Kim, W. R. Gutekunst, L. Song, R. Ramprasad, *Chemistry of Materials* **2020**, *32*, 24 10489.

[16] Z. Yao, B. Sánchez-Lengeling, N. S. Bobbitt, B. J. Bucior, S. G. H. Kumar, S. P. Collins, T. Burns, T. K. Woo, O. K. Farha, R. Q. Snurr, et al., *Nature Machine Intelligence* **2021**, *3*, 1 76.

[17] R. Gómez-Bombarelli, J. N. Wei, D. Duvenaud, J. M. Hernández-Lobato, B. Sánchez-Lengeling, D. Sheberla, J. Aguilera-Iparraguirre, T. D. Hirzel, R. P. Adams, A. Aspuru-Guzik, *ACS central science* **2018**, *4*, 2 268.

[18] T. White, *arXiv preprint arXiv:1609.04468* **2016**.

[19] M. J. Kusner, B. Paige, J. M. Hernández-Lobato, In *International conference on machine learning*. PMLR, **2017** 1945–1954.

[20] A. C. Van Duin, S. Dasgupta, F. Lorant, W. A. Goddard, *The Journal of Physical Chemistry A* **2001**, *105*, 41 9396.

[21] A. Vashisth, C. Ashraf, W. Zhang, C. E. Bakis, A. C. Van Duin, *The Journal of Physical Chemistry A* **2018**, *122*, 32 6633.

[22] H. Moriwaki, Y.-S. Tian, N. Kawashita, T. Takagi, *Journal of cheminformatics* **2018**, *10*, 1 1.

[23] K. Tangthana-Umrung, Q. A. Poutrel, M. Gresil, *Macromolecules* **2021**, *54*, 18 8393.

[24] D. P. Kingma, J. Ba, *arXiv preprint arXiv:1412.6980* **2014**.
